# Prevalence and predictors of hepatitis B virus (HBV) infection in east Africa: evidence from a systematic review and meta-analysis of epidemiological studies published from 2005 to 2020

**DOI:** 10.1186/s13690-021-00686-1

**Published:** 2021-09-18

**Authors:** Hussein Mukasa Kafeero, Dorothy Ndagire, Ponsiano Ocama, Ali Kudamba, Abdul Walusansa, Hakim Sendagire

**Affiliations:** 1 Department of Medical Microbiology, Faculty of Health Sciences, Habib Medical School, Islamic University in Uganda, P.O. Box 7689, Kampala, Uganda; 2 Department of Medical Microbiology, College of Health Sciences, Makerere University, P.O. Box 7062, Kampala, Uganda; 3 Department of Plant Sciences, Microbiology and Biotechnology, College of Natural Sciences, Makerere University, P.O. Box 7062, Kampala, Uganda; 4 Department of Medicine, College of Health Sciences, Makerere University, P.O. Box 7062, Kampala, Uganda; 5 Department of Human Physiology, Faculty of Health Sciences, Habib Medical School, Islamic University in Uganda, P.O. Box 7689, Kampala, Uganda

**Keywords:** East Africa, Hepatitis B, Prevalence, HBsAg, Predictors

## Abstract

**Background:**

The epidemiology of hepatitis B virus (HBV) in the general population in east Africa is not well documented. In this meta-analysis, we examined 37 full published research articles to synthesise up-to-date data on the prevalence and predictors of the HBV burden for the effective prevention and management of the virus in our region.

**Methods:**

We examined 37 full published research articles found using PubMed, Scopus, African Journal Online (AJOL), and Google Scholar between May and October 2020. Dichotomous data on HBV prevalence and predictors of infection were extracted from the individual studies. The HBV prevalence, test of proportion, relative risk, and I^2^ statistics for heterogeneity were calculated using MedCalc software version 19.1.3. Begg’s tests was used to test for publication bias. Sources of heterogeneity were analysed through sensitivity analysis, meta-regression, and sub-group analysis at 95% CI. *P* < 0.05 was considered significant for all analyses.

**Results:**

The prevalence of HBV was generally high (6.025%), with publications from Kenya (8.54%), Uganda (8.454%) and those from between 2011 and 2015 (8.759%) reporting the highest prevalence (*P* < 0.05). Blood transfusion, scarification, promiscuity, HIV seropositivity, and being male were independent predictors significantly associated with HBV infection (*P* < 0.05), with the male sex being the most strongly associated predictor of HBV infection. Meta-regressions for the pooled HBV prevalence and sample size, as well as the year of publication, lacked statistical significance (*P* > 0.05). Omitting the study with the largest sample size slightly increased pooled HBV prevalence to 6.149%, suggesting that the studies are robust. Begg’s test showed no evidence of publication bias for overall meta-analysis (*p* > 0.05).

**Conclusion:**

The burden of HBV is still high, with the male sex, blood transfusion, body scarification, and HIV seropositivity being potential predictors of infection. Thus, it is important to scale up control and prevention measures targeting persons at high risk.

**Supplementary Information:**

The online version contains supplementary material available at 10.1186/s13690-021-00686-1.

## Background

Hepatitis B virus (HBV) is one of the key etiological agents for liver diseases, including chronic hepatitis, liver cirrhosis, and liver cancer [1]. It is the second commonest human carcinogen after tobacco [2]. The virus is highly contagious and is 50 to 100 times more infectious than the human immunodeficiency virus (HIV). Its extreme resilience allows it to survive for more than a week on dry surfaces, complicating its epidemiology and increasing the risk of horizontal intra-familial transmission [3]. According to the World Health Organization (WHO), countries of Africa, Asia, and South America have carrier rates as high as 8%, with sub-Saharan Africa accounting for 20% of the global burden [4]. This implies an unmet need in the control and management of the pandemic in Africa. Furthermore, although a highly effective HBV vaccine is available, immunisation among adult populations in sub-Saharan African countries is neither free nor universal [5–8].

In east Africa, data on the seroprevalence of HBV are isolated and confined to only sub-group studies including, but not limited to, studies of blood donors [9], health care workers [10], pregnant women on antenatal care [11, 12], as well as HIV-positive cohorts [13]. Studies that examine HBV prevalence at national and regional revels are scant in the literature. This means that the risk factors to HBV infection have not been comprehensively evaluated because specific groups may present with unique predictors of infection. Therefore, understanding the epidemiology of HBV on a wide regional scale would not only offer evidence-based data on HBV prevalence but also provide an array of the putative risk factors associated with HBV infection at a regional level.

The risk factors of HBV infection depend largely on beliefs and cultural practices, both of which vary from one community to another [14]. Several primary studies elsewhere have highlighted the key risk factors associated with HBsAg seropositivity, including history of blood transfusion, low level of education, surgery, sexually transmitted infections, abortions, higher mean parity, engaging in early sexual activities, polygamy, being male, having a rural birthplace, and engaging in sex with multiple partners [15–19]. However, Lawal et al. [20] did not observe a significant association between HBV infection and the sharing of a toothbrush, the sharing of needles, incision marks/tattoos, hepatitis B immunisation status, history of blood transfusion, previous surgical operations, sexual exposure/abuse, history of jaundice, or genital circumcision. Thus, because of these inconsistent findings in the literature about which risk factors are more significant for community infections with HBV, our systematic review and meta-analysis aimed to find the key putative predictors associated with the high HBV prevalence in East Africa. This could significantly contribute towards the achievement of the ambitious sustainable development goal of eliminating hepatitis B virus (HBV) infections by 2030 [21]. To the best of our knowledge, this is the first study to give a detailed analysis of the status of HBV infection in our region. The overall pooled prevalence in the region, the sub-group pooled prevalence, and the associated risk factors could be valuable for designing and implementing public health measures to reduce the burden of HBV infection in east Africa.

## Materials and methods

### Systematic review protocol registration, information sources, and search strategies

Our study was designed to investigate the prevalence and predictors of hepatitis B virus infection in east Africa. We registered the protocol with the International Prospective Register of Systematic Reviews (PROSPERO), University of York Centre for Reviews and Dissemination (https://www.crd.york.ac.uk/PROSPERO), under the registration number CRD42021251974. The findings of the review were reported based on the Preferred Reporting Items for Systematic Review and Meta-Analysis (PRISMA) 2020 statement checklist [22]. We searched the following electronic databases; PubMed, Scopus, African Journals Online (AJOL), and Google Scholar for studies published from 2005 to 2020 that investigated the prevalence and predictors of HBV infection in east Africa.

A thorough review of the titles, abstracts, and full papers was done by three reviewers (HMK, AW, and AK). Any disagreement between the three was settled by consensus during weekly evaluation meetings. A full-text analysis of studies that qualified, including identification of duplicated records, was conducted by HMK, AW, and AK. Only the full-text articles were retained for data extraction.

The population, exposure, comparison and outcome (PECO) strategy was used throughout our search strategy. Regarding the population, all studies conducted on the HBV infection status that used study subjects within the east African region constituting the political East African Community (EAC) member states were searched for data extraction. Pertaining to the exposure status, the number of persons exposed and unexposed to HBV within the sample used in the separate studies indicated the prevalence of HBV. Regarding comparison, the data on the predictors of infection were searched for from the main body of the research article and then compared among the exposed and the unexposed to HBV. Finally, concerning the outcome, the central outcome was HBV infection in the population, while the supplementary outcomes were the relative HBsAg seropositivity depending on the diagnostic methods used for HBV detection (ELISA, RDTs, EIA, and CMEA) and the predictors of HBV infection.

The search terms used are presented in Table 1. These were used either in isolation or in combination using the Boolean operators ‘OR’ and ‘AND’. The titles and abstracts were searched for the keywords for HBV prevalence, while the full text of each article was searched for the keywords related to the associated risk factors for HBV infection. The three authors (HMK, AW, and AK) separately extracted the following data: first author, year of publication, country, sampling technique, sample size, hepatitis B surface antigen positivity (HBsAg+), HBsAg detection methods, and quality score.

**Table 1:** Keywords used for searching in databases

Field searched	Key words
HBV prevalence	“Hepatitis B prevalence”, “Hepatitis B prevalence in East Africa”, “Hepatitis B prevalence in Uganda”, “Hepatitis B prevalence in Kenya”, “Hepatitis B prevalence in South Sudan”, “Hepatitis B prevalence in Rwanda”, “Hepatitis B prevalence in Tanzania”, “Hepatitis B prevalence in Burundi”
Risk factors to HBV infection	HBV prevalence and “age”, “Gravidity”, “Marital status” “History of STIs”, “History of STIs”, “History of STIs”, “Education level,” “History of blood transfusion,” “History of scarification,” “Alcohol use,” “HIV sero-status,” “History of body piercing” “History of surgery,” “Number of sexual partners,” “Employment” “Gender”

### Eligibility criteria and study selection

Records were included in the systematic review and meta-analysis if they were full-text case-control or cohort study designs testing for HBsAg, conducted in EAC member states, and published in English in peer-reviewed journals between January of 2005 and October 15th, 2020. Excluded were case reports; reviews; abstracts of conferences; studies with insufficient or inaccessible data; pre-prints; studies with a sample size < 100; studies that never described their sampling technique or that used purposive sampling; studies that investigated other types of viral hepatitis (A, C, D, or E); studies in languages other than English; and studies published before 2005 or after November 30th, 2020.

### Quality assessment and data management

For quality assessment, the Newcastle-Ottawa Scale (NOS) was used [23]. The three dimensions of selection, comparability, and exposure were considered, as described in the scale. For our meta-analysis, the selection assessed the representativeness of the sample, the sample size, the inclusion criteria, and the scope of the study. Comparability assessed the inclusion of HBV infection risk factors and compared them among the study groups. Assessment of inclusion of exposure in the primary studies was done by looking at the outcome of the study, and a statistical tool used for the analysis (Supplementary material, S12; Table A). According to the scale, studies with scores of 9–8 were considered very high quality; 7–6, high quality; and 5–4, moderate quality. Those with scores ≤3 were considered unsatisfactory and were rejected. The three reviewers (HMK, AW, and AK) independently assessed the articles for their overall methodological quality. Data from the articles or their abstracts were entered into the spreadsheet daily by all three authors (HMK, AW, and AK), who compared their separate records on a weekly basis to remove the duplicates. Each of the primary studies was evaluated by three independent hepatitis B virus experts (HMK, AK, and AW) throughout each phase of the review (screening, eligibility, and inclusion in meta-analysis). From each study, the following data were extracted: first author, year of publication, country, sampling technique, sample size, hepatitis B surface antigen positivity (HBsAg+), detection method, quality score (QS), and the predictors of HBV infection.

### Risk of bias in individual studies

For the selection bias, we reviewed the data collection procedures in the primary studies (retrospective or prospective), study design (cohort, cross-sectional, or case-control), and recruitment strategy (from the community or from the hospital). For the information bias, we extracted information on methods used to determine HBV serostatus (rapid diagnostic tests, ELISA, or other immune assays). When more than one method was used to detect HBV serostatus, data were extracted using the best method used, as determined by the evaluator. All studies that used purposive sampling techniques were omitted to avoid biasing the results.

### Publication bias and data synthesis

The publication bias was assessed using Begg’s test, which uses Kendall’s rank correlation coefficient between the meta-analysis effect size and the study weight [24]. Funnel plots were drawn for checking the existence of overall publication bias. The funnel plot asymmetry between the effect size and the meta-analysis study weight was determined by Begg’s test. In this test, *P* > 0.05 indicated no evidence of publication bias (not significant). The test I^2^ statistic was used to assess the heterogeneity among studies. Sources of heterogeneity were analysed through sub-group analysis, sensitivity analysis, and meta-regression. Pooled proportions, test of proportions, relative risk (RR), and the corresponding 95% CI were used to assess the burden of HBV in the region. As a result of high heterogeneities (I^2^ > 73% and P het < 0.05), the random-effects model (REM) was used to pool the HBV prevalence. However, heterogeneity was reduced for some studies that evaluated the relative risk of HBV infection, and in this case, the fixed-effects model (FEM) was used [25]. All the calculations were done using the Medcalc software version 19.1.3. The analyses of HBV prevalence, infection risk factors, and heterogeneity among studies were done at 95% CI, and a *p* < 0.05 was considered significant. Some representative results were presented graphically using forest plots. The prevalence of HBV in each study in the forest plot is indicated by a blue square. The size of the square represents the weight contributed by each study in the meta-analysis. The pooled prevalence for both FEM and REM is shown by the blue diamond.

## Results

### Study identification

**Fig. 1:**
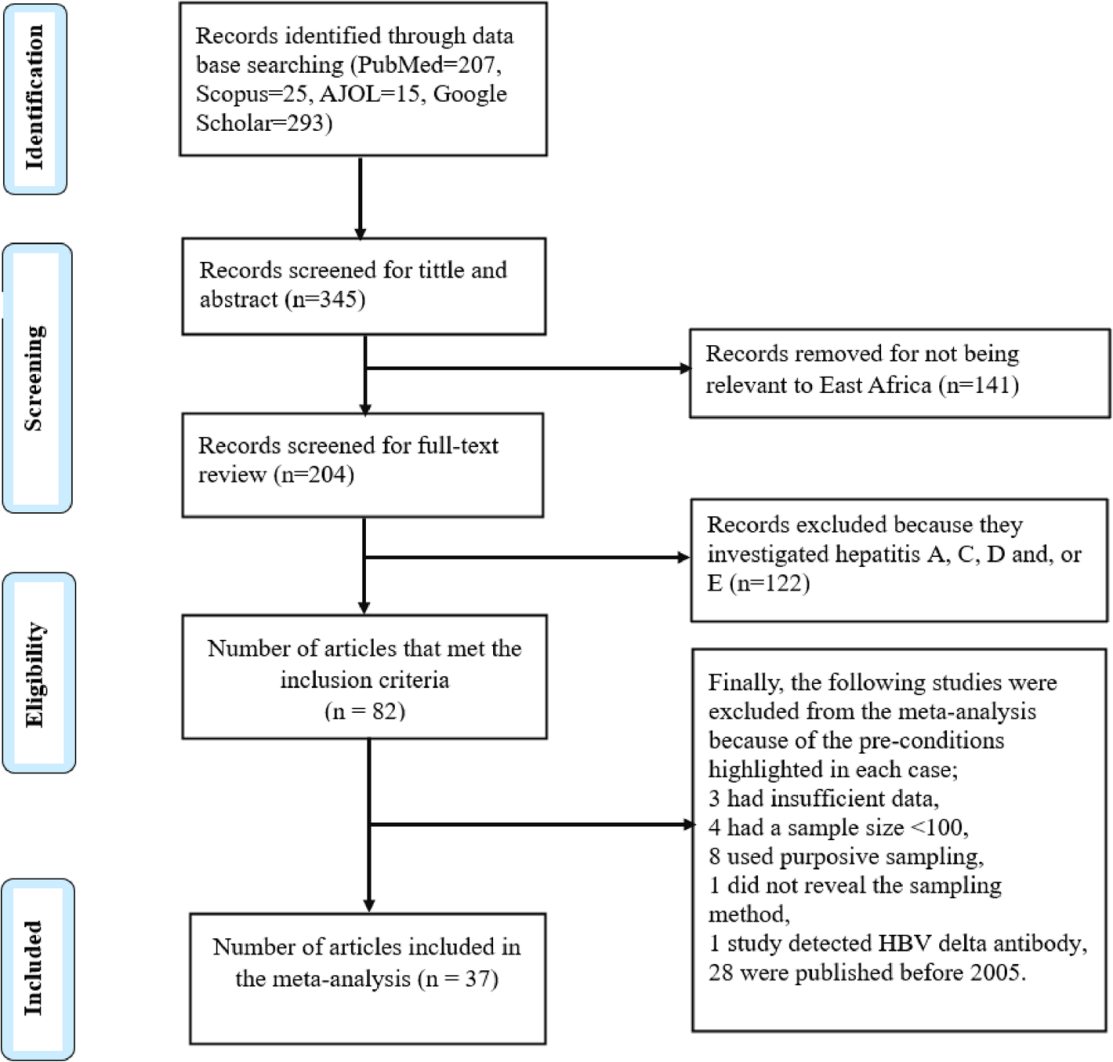
Flow chart for study eligibility following PRISMA criterion; AJOL: African Journal Online, HBV: Hepatitis B Virus

This systematic review and meta-analysis used electronic databases to find published studies on the prevalence and predictors of HBV in east Africa. We found a total of 550 published articles. From these, 345 duplicate records were removed, and 141 articles were excluded after screening of the title and abstract because they were not relevant to east Africa. After this, a total of 204 full-text papers were assessed for eligibility based on the aforementioned inclusion and exclusion criteria. Out of these, 122 records were excluded because they investigated hepatitis A virus (HAV), hepatitis C virus (HCV), hepatitis D virus (HDV), or hepatitis E virus (HEV); 3 were excluded because they had insufficient data; 4, because they had a sample size < 100; 8, because they used purposive sampling; 1, because it did not describe its sampling method; 1, because it detected HBV delta antibodies; 28, because they were published before 2005; and 1, because it was a pre-print. Thirty-seven studies were included in the final quantitative meta-analysis (Fig. 1). They had a total sample size of 525,955 (five hundred twenty-five thousand nine hundred and fifty-five), and included 22,156 (twenty-two thousand one hundred and fifty-six) hepatitis B surface antigen positive (HBsAg+) cases.

### The characteristics of the studies included

**Fig. 2:**
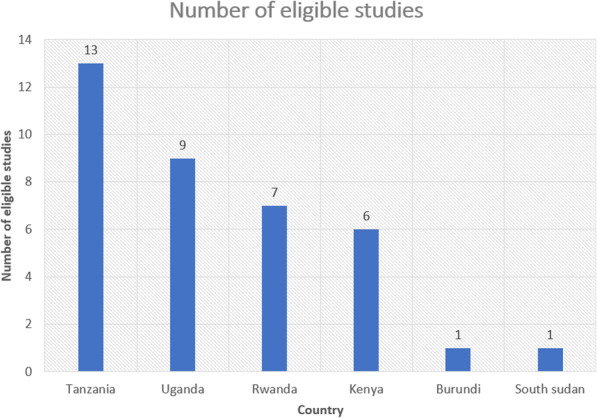
Contribution of eligible studies for inclusion in the meta-analysis by country

**Fig. 3:**
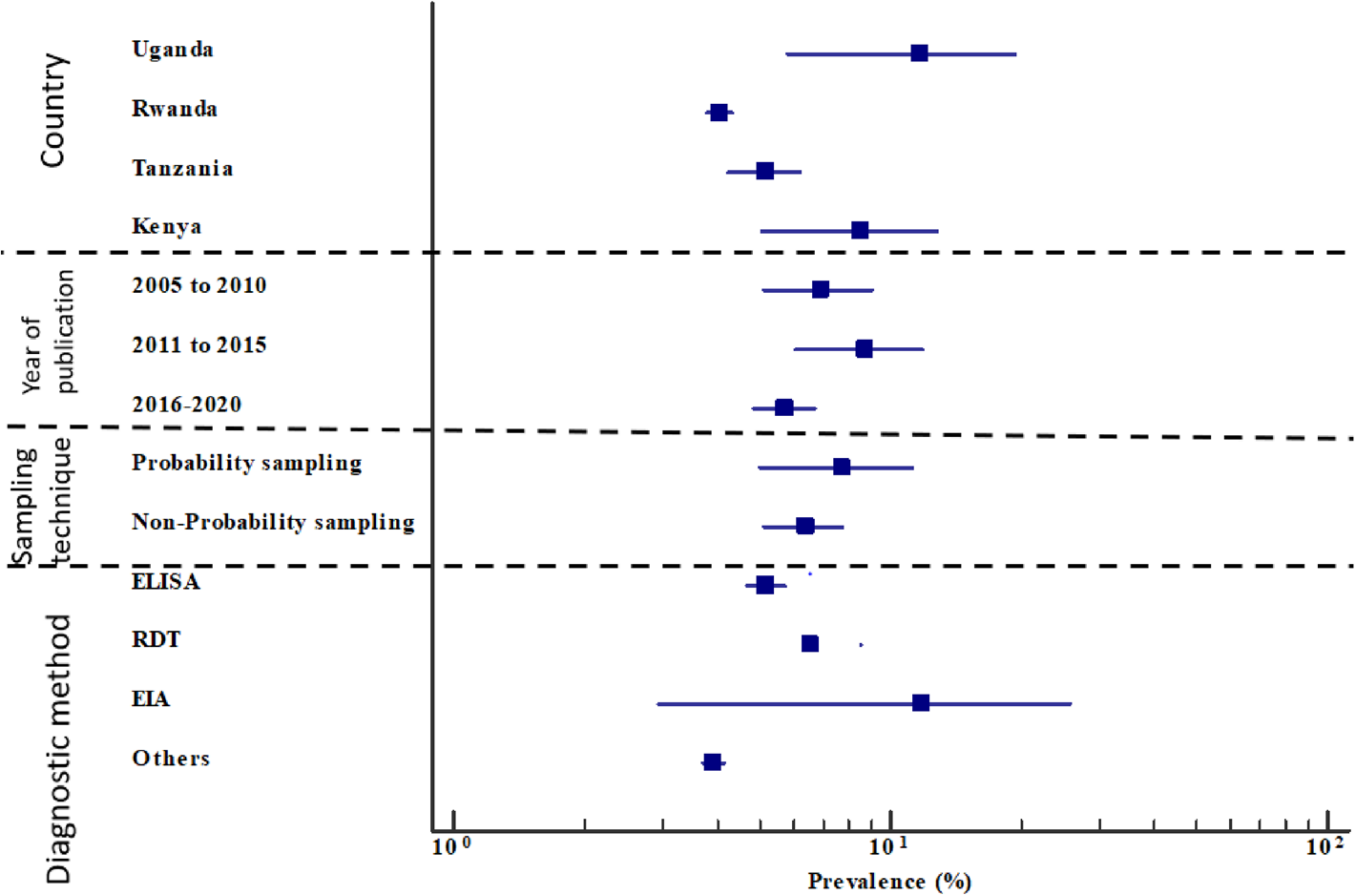
Forest plot showing the HBV prevalence by country, publication period, sampling technique and HBsAg detection method; the blue diamond indicates the pooled prevalence of HBV and the horizontal line shows the 95% confidence interval. The longer the lines, the greater is the deviation from the pooled prevalence; ELISA: Enzyme Linked Immunosorbent Assay, RDT: Rapid Diagnostic Test, EIA: Enzyme Immunoassay

The characteristics of the eligible studies included in our meta-analysis are shown in Table 2. In brief, of the 37 eligible studies, the largest group was done in Tanzania (13/37, 35.1%) with a total sample size of 24,188; followed by Uganda (9/37, 24.3%) with a sample size of 17,103; Rwanda (7/37, 18.9%) with a sample size of 472,532; and Kenya (6/37, 16.2%) with a sample size of 2668; while Burundi and South Sudan had one eligible study each, with sample sizes of 8933 and 280, respectively (Figs. 2 and 3, Table 2). Of the 37 eligible studies, Pirillo et al. [40] had the smallest sample size (of 164), while Makuza et al. [37] had the largest sample size (327,360). Most studies (26/37, 70.3%) with a total sample size of 188,090 used non-probability sampling, while studies that used probability sampling (11/37, 29.7%) had a sample size of 337,865. Most of the eligible studies used rapid diagnostic tests (RDTs) (15/37, 40.4%) and enzyme-linked immunosorbent assay (ELISA) (14/37, 37.8%), with sample sizes of 25,411 and 455,954, respectively. Isolated studies used enzyme immunoassay (EIA) for detection of HBsAg (4/37, 10.8%), with a sample size of 17,065. Other studies (4/37, 10.8%) used the Abbott ARCHITECT system [26], chemiluminescent enzyme immunoassay (CLEIA) [28], and the microparticle enzyme immunoassay (MEIA) [45, 56], with respective sample sizes of 13,121; 21,337; and 587 (Table 2, Fig. 3). Most of the eligible studies included in our meta-analysis were published between 2016 and 2020 (19/37, 51.4%), with a sample size of 494,748, followed by those published between 2011 and 2015 (10/37, 27.0%), with a sample size of 22,021. The rest were published between 2005 and 2010 (8/37, 21.6%), with a sample size of 9180 (Table 2, Fig. 3).

**Table 2:** Characteristics of the included studies in the systematic review and meta-analysis for the prevalence of hepatitis B virus in East Africa

First author, Year	Year	Country	Sampling technique	Sample	HBsAg+	Detection method	QS
Mutagoma et al., 2017 [26] (1)	2017	Rwanda	Entire	13,121	486	AAS	9
Hasegawa I et al., 2006 [27] (2)	2006	Tanzania	voluntary	457	22	CLEIA	7
Twagirumugabe et al., 2017 [28] (3)	2017	Rwanda	Entire	13,637	559	CMIA	8
Bwogi et al., 2009 [29] (4)	2009	Uganda	Probability	5875	606	EIA	9
Kwizera R et al., 2018 [9] (5)	2018	Burundi	Entire	8993	94	EIA	7
Matee et al., 2006 [30] (6)	2006	Tanzania	Entire	1599	141	EIA	8
Mueller et al., 2015 [31] (7)	2015	Tanzania	Entire	598	42	EIA	8
Bayo P, et al., 2014 [32] (8)	2014	Uganda	Probability	402	47	ELISA	8
Bongomin P et al., 2005 [33] (9)	2005	Uganda	Entire	170	19	ELISA	8
Harania RS et al., 2008 [34] (10)	2008	Kenya	Entire	378	23	ELISA	7
Iradukunda et al., 2020 [35] (11)	2020	Rwanda	Random	374	24	ELISA	8
Kirbak et al., 2017 [36] (12)	2017	South Sudan	Probability	280	31	ELISA	8
Makuza et al., 2019 [37] (13)	2019	Rwanda	Probability	327,360	12,865	ELISA	9
Muriuki et al., 2013 [38] (14)	2013	Kenya	Probability	300	21	ELISA	7
Ngaira et al., 2016 [39] (15)	2016	Kenya	Entire	287	11	ELISA	7
Pirillo et al., 2007 [40] (16)	2007	Uganda	Entire	164	8	ELISA	7
Rachel et al., 2018 [13] (17)	2018	Uganda	Entire	8042	359	ELISA	8
Rusine et al., 2013 [41] (18)	2013	Rwanda	Voluntary	402	21	ELISA	7
Telatela SP et al., 2007 [42] (19)	2007	Tanzania	Entire	167	2	ELISA	7
Umutesi et al., 2017 [43] (20)	2017	Rwanda	voluntary	117,258	5042	ELISA	7
Ziraba et al., 2010 [44] (21)	2010	Uganda	Entire	370	30	ELISA	8
Rashid S et al., 2014 [45] (22)	2014	Tanzania	Consecutive	310	12	MEIA	7
Bartonjo, G et al., 2019 [46] (23)	2019	Kenya	Probability	594	33	RDT	8
Geffert et al., 2020 [47] (24)	2020	Tanzania	Entire	723	22	RDT	7
Hawkins C et al., 2013 [48] (25)	2013	Tanzania	Entire	17,539	1079	RDT	8
Kamenya et al., 2017 [49] (26)	2017	Tanzania	Entire	300	7	RDT	8
Kapinga, D. R. 2017 [50] (27)	2017	Tanzania	Random	600	31	RDT	8
Kateera F et al., 2015 [10] (28)	2015	Rwanda	Entire	371	11	RDT	8
Kayondo SP et al., 2019 [11] (29)	2019	Uganda	Probability	340	10	RDT	8
Kilonzo SB et al., 2017 [51] (30)	2017	Tanzania	Entire	743	49	RDT	7
Kerubo G et al., 2015 [52] (31)	2015	Kenya	voluntary	268	56	RDT	7
Kerubo G et al., 2015 [52] (31)	2015	Kenya	voluntary	1041	117	RDT	7
Mirambo MM et al.,2016 [8] (32)	2016	Tanzania	Entire	211	8	RDT	8
Ng’wamkai et al., 2019 [53] (33)	2019	Tanzania	Entire	499	29	RDT	8
Ochola et al., 2013 [6] (34)	2013	Uganda	Probability	790	139	RDT	8
Shao R, et al., 2018 [54] (35)	2018	Tanzania	Entire	442	25	RDT	8
Chiesa et al., 2020 [55] (36)	2020	Uganda	Probability	950	75	RDT	8

*EIA* Enzyme Immunoassay, *ELISA* Enzyme Linked Immunosorbent Assay, *RDT* Rapid Diagnostic Test, *CMIA* Chemiluminescent Micro-particles Immunoassay, *MEIA* Micro-particle Enzyme Immunoassay, *CLEIA* Chemiluminescent enzyme Immunoassay, *QS* Quality score, *HBsAg* Hepatitis B Surface Antigen

### Prevalence of hepatitis B in the east Africa region

**Fig. 4:**
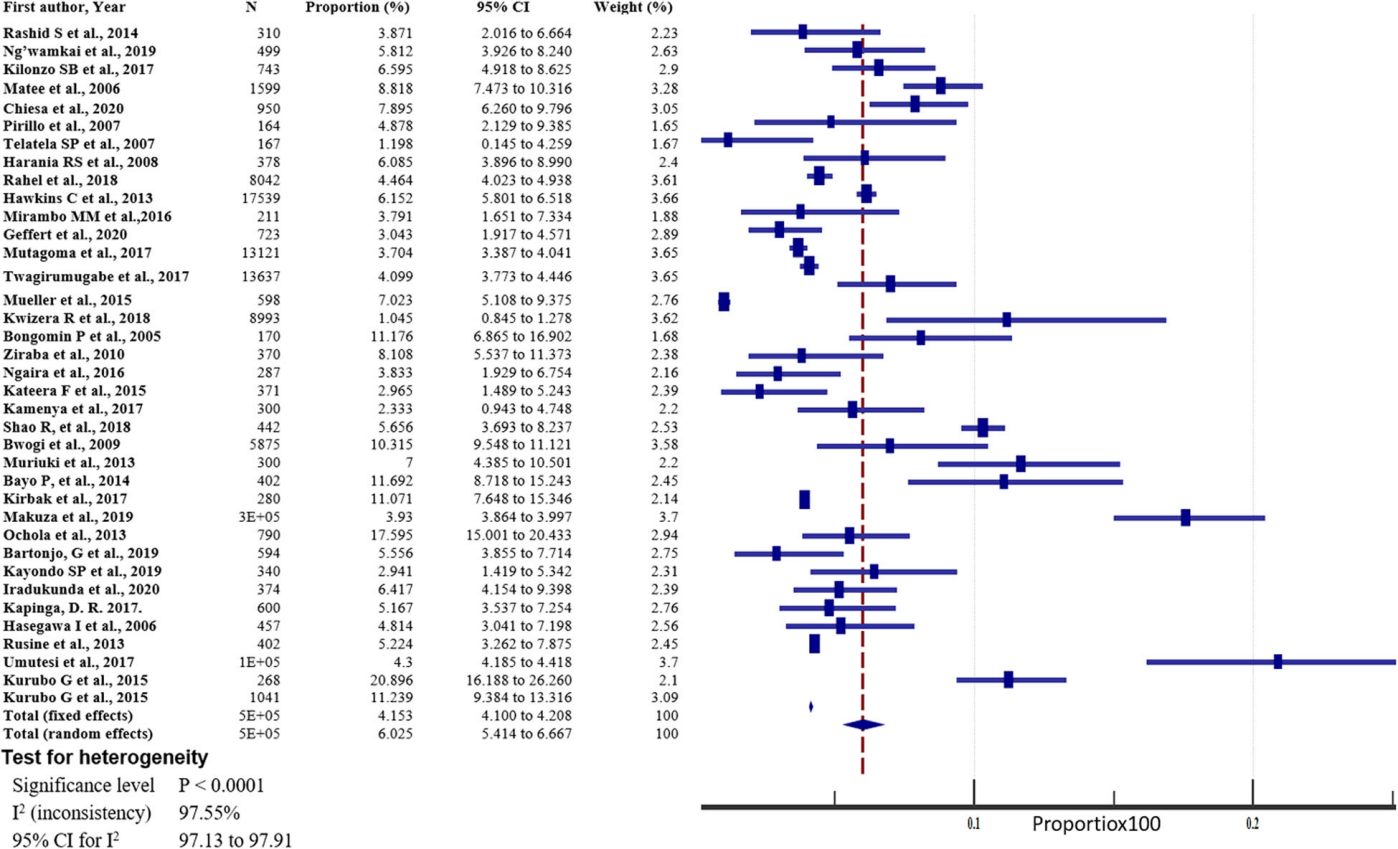
Pooled prevalence estimate of HBV in East-Africa by random effects model

**Fig. 5:**
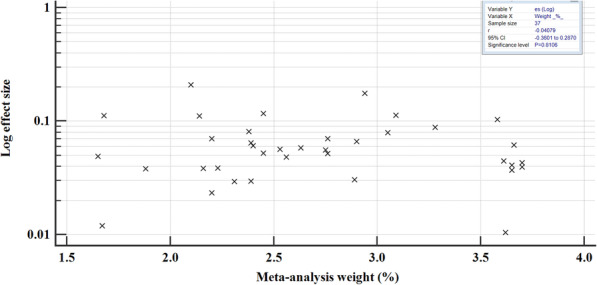
Begg’s correlation test for publication bias; correlation of log effect size with meta-analysis weight for all the epidemiological studies published between 2005 to 2020 included in the meta-analysis

**Fig. 6:**
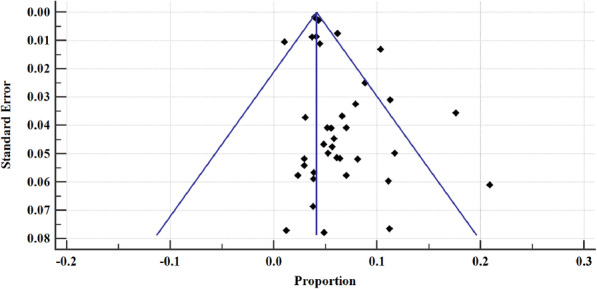
Bias assessment plot of studies reporting hepatitis B virus prevalence in East Africa from epidemiological studies published between 2005 to 2020 included in the data synthesis

The prevalence of hepatitis B in east African between 2005 and 2020 varied, widely ranging from 1.05% (95% CI = 0.845 to 1.278%) [54] to 20.896% (95% CI = 16.188 to 26.260%) [52] (Fig. 4). The overall HBV pooled prevalence among the sample of 525,955 (five hundred twenty-five thousand nine hundred and fifty-five) and 22,156 (twenty-two thousand one hundred and fifty-six) cases was 6.025% (95% CI = 5.414 to 6.667%) with a heterogeneity (I^2^) of 97.55% (*P* > 0.0001) (Fig. 4). The Begg’s test demonstrated no evidence of publication bias among the analysis (*p* = 0.8106) (Fig. 5), despite a mild funnel plot asymmetry (Fig. 6).

### Meta-analysis of subgroups

As shown in Table 3 and Fig. 3, we subdivided our meta-analysis into groups, which included the country where the study was conducted, the year of publication, the sampling technique, and the HBsAg diagnostic technique. Burundi and South Sudan were left out from sub-group analyses because each country had only one eligible study. In all the sub-group analyses, the heterogeneity remained high (I^2^ > 89%, *p* < 0.0001), but was lower for ‘other diagnostic tests’ besides ELISA, RDT, and EIA (I^2^ = 22.55%, *p* = 0.257).

**Table 3:** Subgroup analysis of the HBV pooled prevalence estimation in East Africa from 2005 to 2020

Sub-groups			Analysis of HBV prevalence		Analysis of heterogeneity		
Variable	Category	No	Prevalence %(95%CI)	*P* value	I^2^% (95%CI)	P het	Model
	Kenya	**6**	8.54 (5.09 to 12.76) Ref		92.36 (86.11 to 95.79)	< 0.0001	Random
Country	Uganda	9	8.454 (5.76 to 11.73)	0.9637	97.28 (96.15 to 98.08)	< 0.0001	Random
	Rwanda	7	4.06 (3.80 to 4.34)	< 0.0001*	85.39 (71.83 to 92.42)	< 0.0001	Random
	Tanzania	13	5.16 (4.24 to 6.17)	0.0006*	80.65 (67.84 to 88.35)	< 0.0001	Random
Year of Publication	2011–2015	10	8.759 (6.09 to 11.85) Ref		95.65 (93.65 to 97.2)	< 0.0001	Random
	2005–2010	8	6.95 (5.11 to 9.05)	0.1208	87.4 (77.34 to 92.98)	< 0.0001	Random
	2016–2020	19	4.388 (3.901 to 4.903)	< 0.0001*	96.17 (95.04 to 97.04)	< 0.0001	Random
Sampling Technique	Probability	11	7.799 (5.182 to 10.897)		98.45 (97.99 to 98.80)	< 0.0001	Random
	Non-probability	26	5.361 (4.567 to 6.215)	< 0.0001*	96.91 (96.21 to 96.49)	< 0.0001	Random
Diagnostic Technique	ELISA	14	5.214 (4.71 to 5.74) Ref		90.48 (85.79 to 93.62)	< 0.0001	Random
	RDT	15	6.638 (4.997 to 8.493)	0.0126*	94.18 (91.88 to 95.83)	< 0.0001	Random
	EIA	4	6.127 (1.259 to 14.303)	0.2349	99.62 (99.50 to 99.72)	< 0.0001	Random
	Others	4	3.924 (3.697 to 4.16)	0.0622	22.55 (0.00 to 90.00)	0.257	Fixed

*ELISA* Enzyme Linked Immunosorbent Assay, *RDT* Rapid Diagnostic Test, *EIA* Enzyme Immunoassay

*P value of sub group prevalence with respect to the group reference (where applicable) < 0.05 statistically significant

By country, Kenya had the highest prevalence of 8.54% (95% CI = 5.09 to 12.76%) from 6 studies closely followed by Uganda with a pooled prevalence of 8.454% (95%CI = 5.76 to 11.73%) from 9 studies and then Tanzania with a prevalence of 5.16% (95% CI = 4.24 to 6.17%) from 13 studies. The lowest HBV prevalence was registered in Rwanda, with 4.06% (95% CI = 3.80 to 4.34%) among 7 studies (Supplementary materials S1-S4, Figures A-D).

Regarding the year of publication, the lowest pooled prevalence was 4.388% (95%CI = 3.901 to 4.903%) found in 19 studies published between 2016 and 2020, closely followed by a prevalence of 6.95% (95% CI = 5.11 to 9.05%) from 8 studies published between 2005 and 2010. The highest prevalence of 8.759% (95% CI = 6.09 to 11.85%) was pooled from 10 published studies between 2011 and 2015 (Supplementary Materials S5–7, Figures E-G).

Pertaining to the sampling technique, the pooled prevalence among the 26 studies that used non-probability methods was 5.361% (95%CI = 4.567 to 6.215%), while the pooled prevalence for the 11 eligible studies that used probability methods was 7.799% (95%CI = 5.182 to 10.897%). Concerning HBsAg detection method, the 14 studies that used ELISA reported a prevalence rate of 5.214% (95%CI = 4.71 to 5.74%), the 15 studies that used RDT reported a prevalence rate of 6.638% (95%CI = 4.997 to 8.493%) and the 4 studies that used EIA reported a pooled prevalence of 6.127% (95%CI = 1.259 to 14.303%). The studies that used other diagnostic tests (MEIA, CLEIA, and AAS) reported a pooled HBV prevalence of 3.924% (95% CI = 3.697 to 4.16%) (Supplementary Materials S8–11, Figures H-K).

When we analysed the relative prevalence of HBV by country, year of publication, sampling technique, and method of HBsAg detection, Kenya and Uganda had significantly higher HBV pooled prevalence than Tanzania and Rwanda (*P* < 0.05). Similarly, articles published from 2011 to 2015 had a significantly higher HBV prevalence than those published from 2005 to 2010 and from 2016 to 2020 (*P* < 0.05). Further still, studies that detected the HBsAg using RDT reported significantly higher HBV prevalence than those that used ELISA (Table 3).

### Predictors of HBV infection in east Africa

**Fig. 7:**
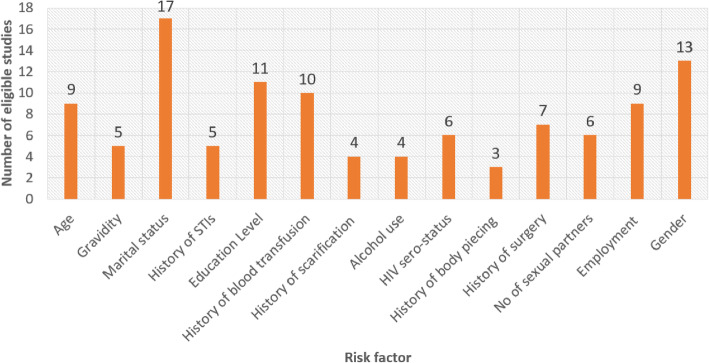
Number of studies with disaggregated data on risk factors associated with HBV infection

Of all the records eligible for inclusion in our meta-analysis, 9 had disaggregated data on age (< 20 or ≥ 20 years); 5, on gravidity (primagravida or multigravida); 17, on marital status (married or unmarried); 5, on history of sexually transmitted infections (prior infection or no); 11, on education level (≤primary or ≥ secondary); 10, on history of blood transfusion (prior transfusion or no); 4, on history of scarification (body scarification or naïve); 4, on alcohol use (yes or no); 6, on HIV serostatus (HIV+ or HIV−); 3, on history of body piercing (prior body piercing or naïve); 7, on history of surgery (underwent surgery before or not); 6, on number of sexual partners (1 or ≥ 2); 9, on employment (formal or informal); and 13, on sex (male or female) (Fig. 7).

### Meta-analysis of the predictors of HBV infection in east Africa

When we analysed the data that investigated the predictors of HBsAg seropositivity by age, gravidity, marital status, sexually transmitted infection, level of education, blood transfusion, scarification, alcohol use, HIV serostatus, body piercing, surgery, person who have sex with multiple partners, and gender, the results were interesting. The HBV prevalence was significantly associated with multigravidity (3.84%; 95% CI = 3.40 to 4.33%); singleness 8.644% (95%CI = 5.21 to 12.85%); blood transfusion 11.057% (95%CI = 5.84 to 17.68%); scarification 8.102% (95%CI = 4.47 to 12.696%); HIV seropositivity 12.131% (95%CI = 7.92 to 17.52%); surgery (13.2%; 95% CI = 0.003 to 45.2%); having sex with multiple partners 9.53% (95%CI = 5.509 to 14.512%); and being male 8.561% (95%CI = 6.66 to 10.675%) (*P* < 0.05). However, we found no significant association between age, sexually transmitted infections, education level, alcohol use, body piercing, or employment with the risk of HBV infection (*P* > 0.05). For most of the risk factor meta-analyses, heterogeneity remained high (I^2^ > 80%, P_het_ < 0.0001), but it was reduced for multigravidity, history of STIs, secondary level of education, routine alcohol use, HIV seropositivity, and body piercing (I^2^ < 73%, P_het_ > 0.0001) (Table 4).

**Table 4:** Analysis of the risk factors associated with HBV infection in East Africa

			Analysis of HBV prevalence		Analysis of heterogeneity of studies		
Risk factor	Category	Number	Proportion%, (95%CI)	*P* value	I^2^, 95%CI	P het	Model
Age	< 20 years	9	4.796 (1.88 to 8.97)		80.6 (64.07 to 89.54)	< 0.0001	Random
	> 20 years	9	5.74 (3.52 to 8.46)	0.28	89.27 (81.9 to 93.65)	< 0.0001	Random
Gravidity	Primigravida	5	2.84 (1.37 to 4.82)		81.6 (57.40 to 92.06)	0.0002	Random
	Multigravida	5	3.84 (3.40 to 4.33)	0.0006*	32.98 (0.00 to 74.58	0.2015	Fixed
Marital status	Married	17	5.508 (3.35 to 8.17)		98.91 (98.69 to 99.1)	< 0.0001	Random
	Not Married	17	8.644 (5.21 to 12.85)	< 0.0001*	96.98 (96.13 to 97.6)	< 0.0001	Random
History of STIs	Yes	5	7.33 (4.77 to 10.67)		25.7 (0.00 to 70.27)	0.2015	Fixed
	No	5	4.41 (2.79 to 6.39)	0.5858	68.61 (19.22 to 87.8)	0.0126	Random
Education Level	Primary & below	11	6.096 (4.42 to 8.02)		83.49 (71.9 to 90.29)	< 0.0001	Random
	Secondary & above	11	5.917 (4.374 to 7.676)	0.6434	73.11 (50.84 to 85.3)	0.0001	Random
History of blood transfusion	Yes	10	11.057 (5.84 to 17.68)		79.6 (63.2 to 88.72)	< 0.0001	Random
	No	10	4.915 (3.492 to 6.567)	< 0.0001*	89.5 (82.74 to 93.56)	< 0.0001	Random
History of scarification	Yes	4	8.102 (4.47 to 12.696)		89.7 (78.79 to 95.0)	< 0.0001	Random
	No	4	5.049 (3.318 to 7.120)	< 0.0001*	84.4 (65.09 to 93.04)	< 0.0001	Fixed
Alcohol use	Yes	4	7.37 (3.46to 12.6)		64.88 (0.00 to 88.09)	0.036	Random
	No	4	3.48 (2.55 to 4.63)	0.4703	30.01 (0.00 to 74.53)	0.2322	Fixed
HIV sero-status	Positive	6	12.131 (7.92 to 17.52)		12.15 (0.00 to 78.35)	0.3374	Fixed
	Negative	6	5.863 (3.082 to 9.461)	0.0030*	89.81 (80.5 to 94.67)	< 0.0001	Random
History of body piecing	Yes	3	3.813 (2.41 to 5.70)		59.57 (0.00 to 88.48)	0.0843	Fixed
	No	3	4.24 (2.903 to 5.95)	0.9394	0.00 (0.00 to 92.27)	0.6478	Fixed
History of surgery	Yes	7	13.2 (0.003 to 45.2)		99.50 (99.37 to 99.60)	< 0.0001	Random
	No	7	4.85 (1.43 to 10.14)	< 0.0001*	97.32 (96.0 to 38.20	< 0.0001	Random
No of sexual partners	One	6	5.116 (3.124 to 7.566)		89.04 (78.8 to 94.34)	< 0.0001	Random
	More than one	6	9.53 (5.509 to 14.512)	< 0.0001*	87.36 (74.9 to 93.65)	< 0.0001	Random
Employment	Formal	9	7.70 (2.38 to 15.69)		95.65 (93.51 to 97.1)	< 0.0001	Random
	Non formal	9	10.48 (2.72 to 22.5)	0.333	98.6 (98.12 to 98.93)	< 0.0001	Random
Gender	Male	13	8.561 (6.66 to 10.675)		93.53 (90.6 to 95.54)	< 0.0001	Random
	Female	13	6.986 (4.841 to 9.491)	0.0007*	96.63 (95.44 to 97.5)	< 0.0001	Random

*STIs* Sexually Transmitted Infections

*P value of risk factor < 0.05 statistically significant

### Relative risk meta-analysis for hepatitis B virus infection in east Africa

**Fig. 8:**
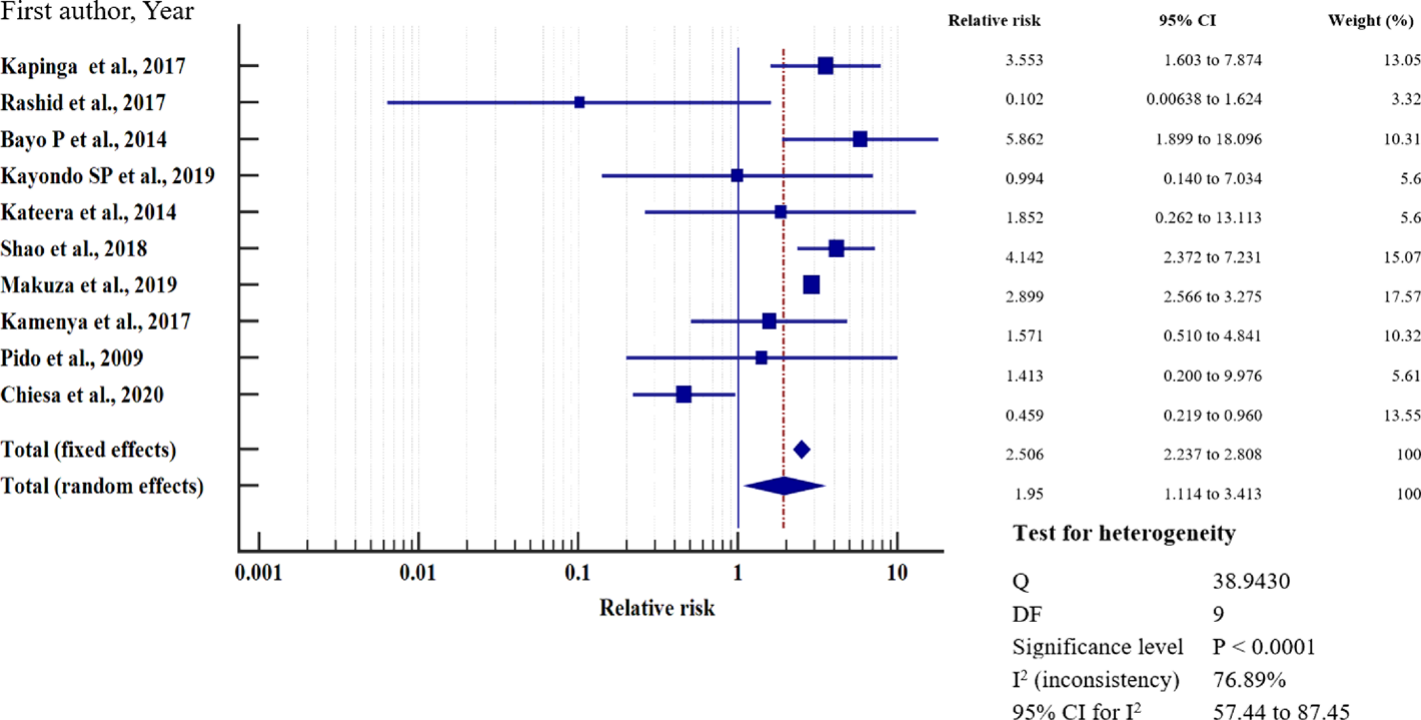
History of blood transfusion on the risk of HBV infection when compared between those who have had blood transfusion and those who have never transfused. The RR > 1 indicates increased risk or susceptibility to HBV infection whereas the RR < 1 indicates reduced risk to HBV infection

**Fig. 9:**
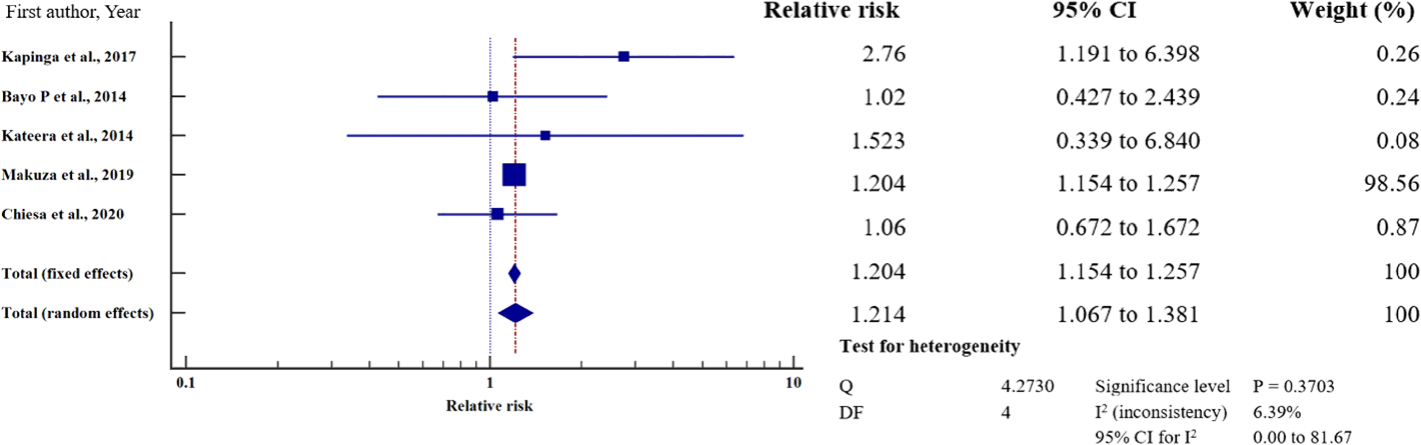
History of scarification on the risk of HBV infection when compared between those with the history of scarification and those who have never scarified. The RR > 1 indicates increased risk or susceptibility to HBV infection whereas the RR < 1 indicates reduced risk to HBV infection

**Fig. 10:**
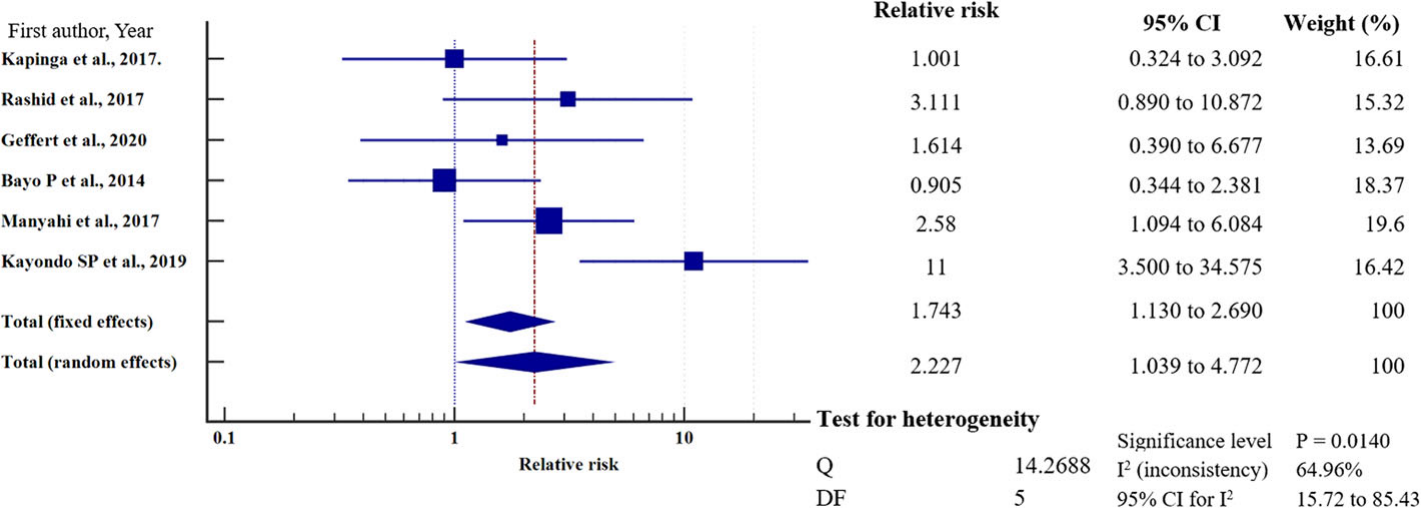
HIV sero-status on the risk of HBV infection when compared between the HIV sero-positive and sero-negative. The RR > 1 indicates increased risk or susceptibility to HBV infection whereas the RR < 1 indicates reduced risk to HBV infection

**Fig. 11:**
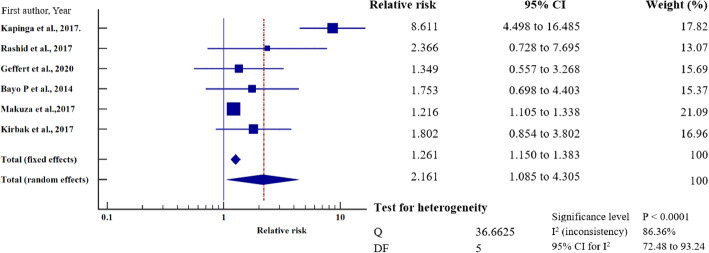
Number of sexual partners on the risk of HBV infection when compared those with one sexual partner and those with multiple sexual partners. The RR > 1 indicates increased risk or susceptibility to HBV infection whereas the RR < 1 indicates reduced risk to HBV infection

**Fig. 12:**
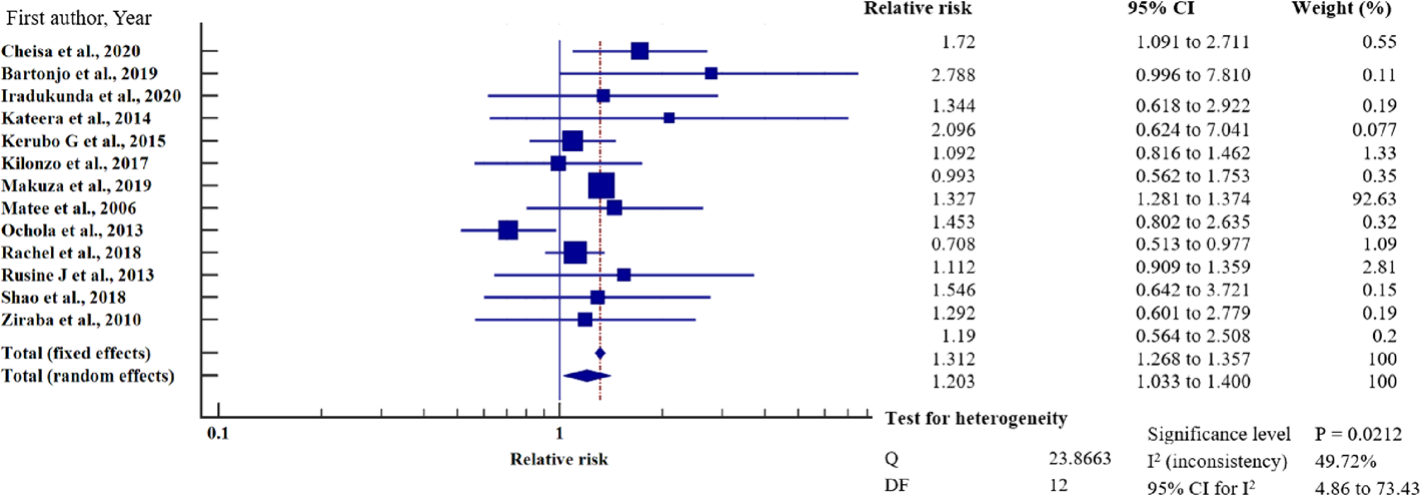
Sex on the risk of HBV infection when compared between the male and female. The RR > 1 indicates increased risk or susceptibility to HBV infection whereas the RR < 1 indicates reduced risk to HBV infection

**Fig. 13:**
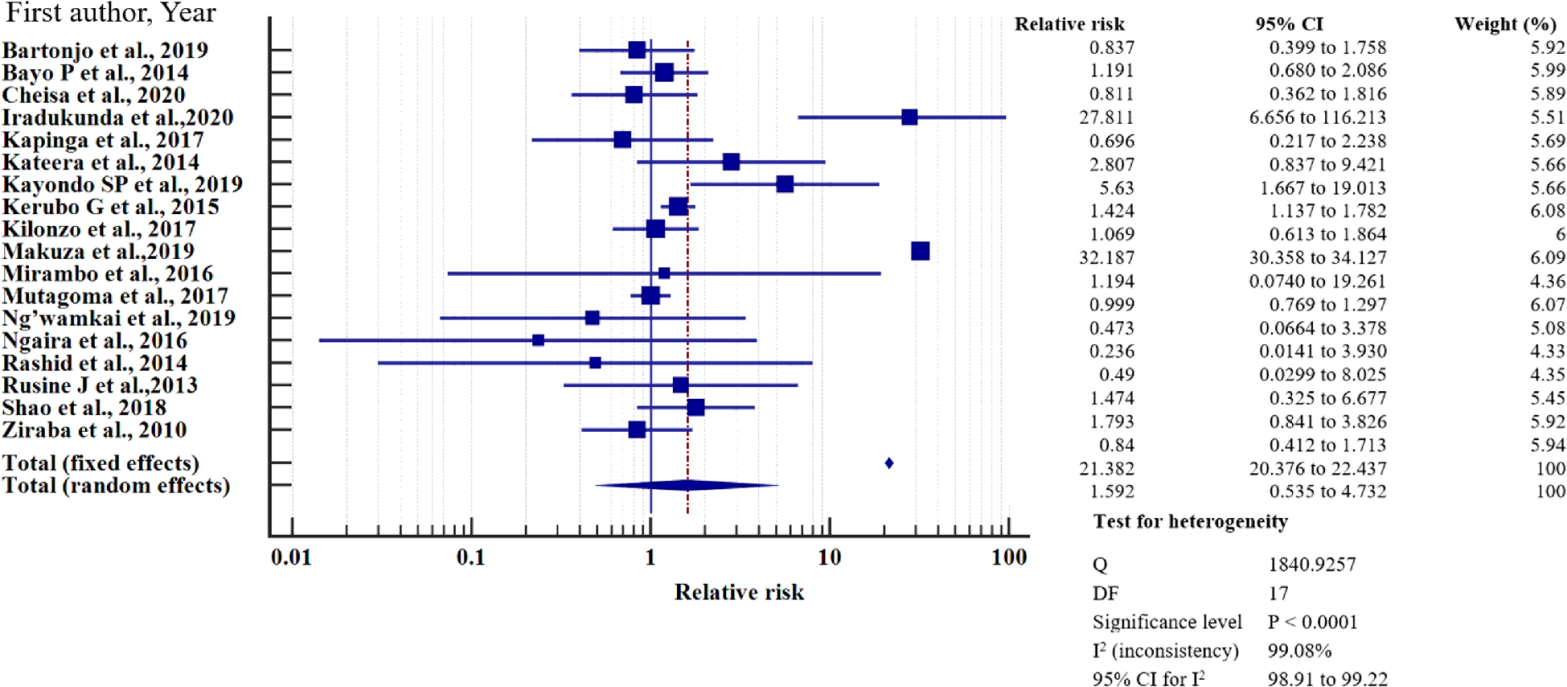
Marital status on the risk of HBV infection when compared between the married and unmarried. Relative Risk (RR) > 1 indicates increased risk or susceptibility to HBV infection whereas the RR < 1 indicates reduced risk to HBV infection

**Fig. 14:**
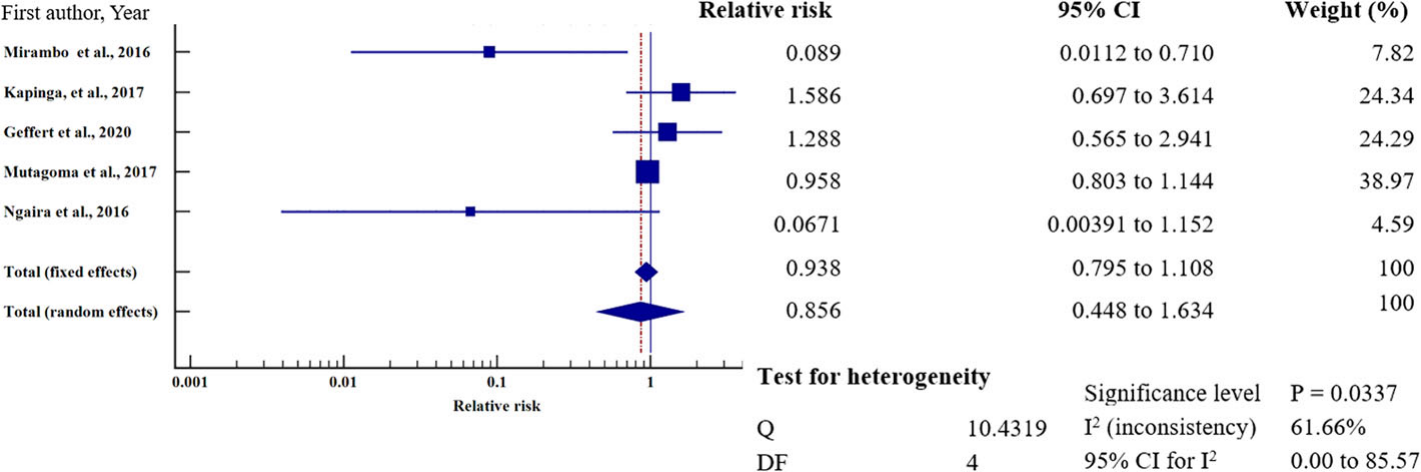
Gravidity on the risk of HBV infection when compared between the primagravida and multigravida. Relative Risk (RR) > 1 indicates increased risk or susceptibility to HBV infection whereas the RR < 1 indicates reduced risk to HBV infection

**Fig. 15:**
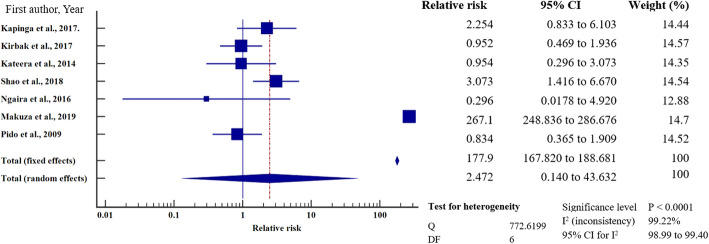
History of surgery on the risk of HBV infection when compared between those previously exposed to surgical operation and those who have never. The RR > 1 indicates increased risk or susceptibility to HBV infection whereas the RR < 1 indicates reduced risk to HBV infection

To investigate the relative risk for HBV infection in east Africa, we meta-analysed the data on the statistically significant predictors of HBV infection, as presented in Table 4. Based on this criterion, eight factors (marital status, gravidity, blood transfusion, scarification, HIV seropositivity, surgery, having sex with multiple partners, and gender) merited relative risk meta-analysis (Table 5). The independent risk factors significantly associated with HBV infection were blood transfusion (relative risk 1.950, 95%CI = 1.114 to 3.413%, *P* = 0.019); body scarification (relative risk 1.204, 95%CI = 1.154 to 1.257%, *P* < 0.001); HIV seropositivity (relative risk 2.227, 95%CI = 1.039 to 4.772%, *P* = 0.039); having sex with multiple partners (relative risk 2.161, 95%CI = 1.085 to 4.305%, *P* = 0.028); and being male (relative risk 1.312, 95%CI = 1.268 to 1.357%, *P* < 0.001) (Table 5, Figs. 8, 9, 10, 11, 12). For most of the analyses, heterogeneity index (I^2^) remained high (> 85%), but it was lower for scarification (I^2^ = 23.31%) and vanished for HIV seropositivity (I^2^ = 0.00%) (Table 5). Conversely, marital status (relative risk 1.592, 95%CI = 0.535 to 4.732%, *P* = 0.403), magnitude of gravidity (relative risk 0.856, 95% CI *=* 0.448 to 1.634%, *P* = 0.637), and surgery (relative risk 2.47, 95% CI = 0.14 to 43.63%, *P* = 0.537) were not significantly associated with the risk of HBV infection (*P* > 0.05) (Table 5, Figs. 13, 14, 15).

**Table 5:** Analysis of the statistically significant risk factors associated with HBV infection in East Africa

	Analysis				
Risk factor	Number	Relative risk %(95%CI)	*P* value	Heterogeneity (I^2^%)	Model
Marriage	18	1.592 (0.535 to 4.732)	0.403	99.08	Random
Gravidity	5	0.856 (0.448 to 1.634)	0.637	61.66	Random
History of blood transfusion	10	1.950 (1.114 to 3.413)	0.019*	76.89	Random
Scarification	4	1.204 (1.154 to 1.257)	< 0.001*	6.39%	Fixed
HIV sero-positivity	6	2.227 (1.039 to 4.772)	0.039*	64.96%	Random
History of surgery	7	2.47 (0.14 to 43.63)	0.537	99.22	Random
≥2 sexual partners	6	2.161 (1.085 to 4.305)	0.028*	86.36	Random
Male sex	12	1.312 (1.268 to 1.357)	< 0.001*	49.72	Fixed

*P value of risk factor < 0.05 statistically significant

### Meta-regression

**Fig. 16:**
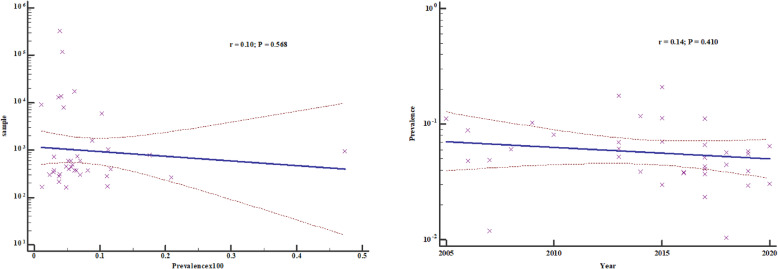
Meta-regression analysis for the heterogeneity of HBV prevalence in East Africa 2005 to 2020

We performed meta-regression analysis on the two continuous variables of sample size and year of publication to assess how they varied with the prevalence of HBV. There was no statistical significance in either regression (*P* > 0.05) (Fig. 16), suggesting that the results are robust and the overall HBV prevalence is not directly influenced by sample size or year of publication.

### Sensitivity analysis

**Fig. 17:**
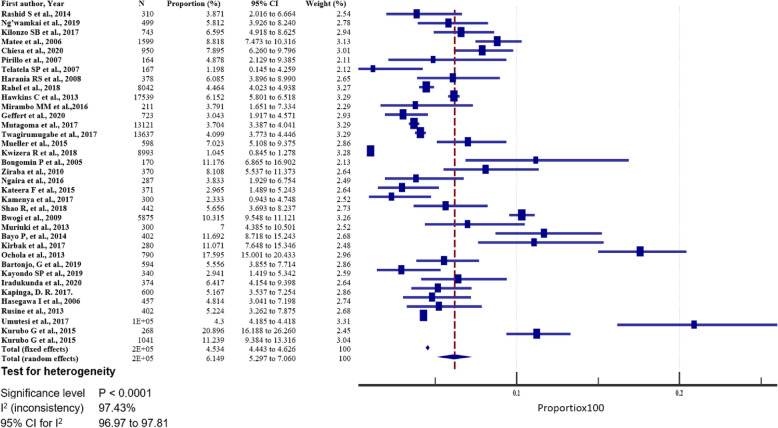
Forest plot for sensitivity analysis with one study with the highest sample size omitted

**Fig. 18:**
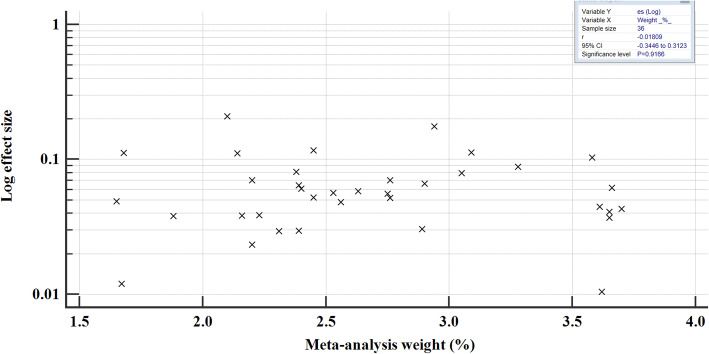
Begg’s correlation test for publication bias; correlation of log effect size and meta-analysis weight after exclusion of the study with the largest sample size

**Fig. 19:**
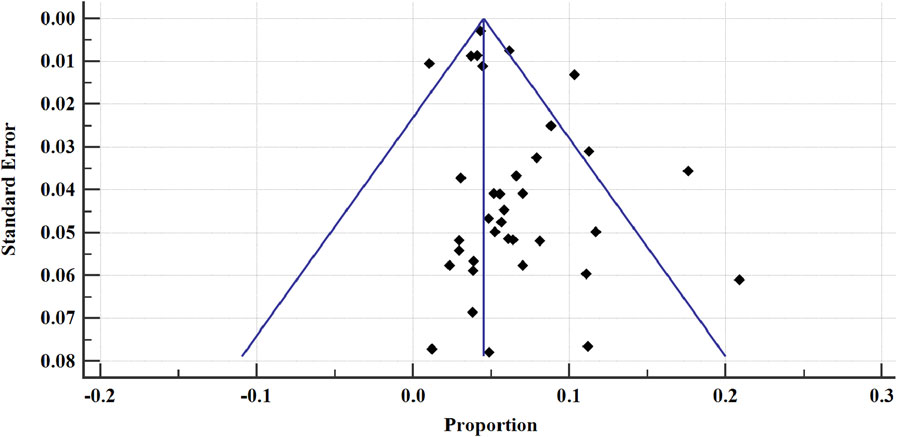
Bias assessment plot for sensitivity analysis of studies reporting hepatitis B virus prevalence in East Africa from 2005 to 2020

For sensitivity analysis, we removed the study with the largest sample size [37]. The overall HBsAg+ pooled prevalence rate before omission was 6.025% (95% CI = 5.414 to 6.667%) with a heterogeneity (I^2^) of 97.55%, *P* > 0.0001. After the omission, the pooled prevalence rate increased slightly to 6.149% (95%CI = 5.297 to 7.06%), with a heterogeneity (I^2^) of 97.43%, *P* < 0.0001. Thus, the calculated pooled prevalence was not affected by a single study, suggesting that the results are robust (Fig. 17). Furthermore, Begg’s correlation test for publication bias after omitting the study did not show evidence of publication bias (*P* = 0.9166) (Figs. 18), despite the mild right skew of the data in the funnel plot (Fig. 19).

## Discussion

In our meta-analysis, almost all the eligible studies were cross-sectional and reflected the HBV prevalence at one point in time, ranging from 1.05 to 20.896%, with a pooled prevalence of 6.025%. In 37.8% of the studies (14/37), the prevalence was higher than the 6.1% reported by the World Health Organization (WHO) in the African region [4]. Thus, HBV is still a significant burden in east Africa, aggravated by the weak health care systems in the region. Furthermore, the burden is a challenge all over Africa because many meta-analyses conducted in other African countries found even higher pooled prevalence than the prevalence reported in the current study. For example, in Nigeria, the prevalence was 13.6% [57]; in Ghana, 12.3% [58]; in Cameroon, 10.6% [59]; in Burkina Faso, 11.21% [60]; in Sudan, 12.07% [61]; and in Somalia, 19.0% [62]. Consequently, the results of our meta-analysis and those of other meta-analyses show that HBV is hyperendemic in many African countries [29] and that a significant budgetary resource allocation by African governments in general and the EAC member states in particular is needed to address the burden.

Pooling the prevalence of HBV by country, Kenya had the highest prevalence rate of 8.54%, followed by Uganda (8.454%) and Tanzania (5.16%), and finally Rwanda with the lowest prevalence (4.1%). The prevalence rates reported in our study for Uganda, Kenya, and Tanzania are in fair conformity with the respective prevalence rates of 9.2, 5.2, and 7.2% reported by Schweitzer A. et al. [63] in their systematic review and meta-analysis on the global prevalence of HBV. The differences in prevalence rates of HBV by country cannot be ascertained exactly, but can probably be explained by differences in infection risk factors [63], vaccination coverage by country [64], endemicity [62], prevention and control measures [65], host genetic factors, and infectivity of the HBV genotypes circulating in the regions [66].

For countries with low prevalence rates, it is plausible that there have been concerted efforts by both the health care workers and community members to improve HBV awareness, as reported in primary studies by Quadri et al. [67] and Shah et al. [68] for Tanzania. On the other hand, for countries with high prevalence rates, it is possible that there has been an increase in high-risk behaviours, such as unprotected sex, tattooing, and traditional scarification, combined with low levels of HBV awareness, as reported in primary studies conducted in Kenya by Awili et al. [69] and Ngaira et al. [42].

The high prevalence of HBV in Uganda has earlier been reported by Ott et al. in their study on time trends of global chronic HBV infection [70]. However, the high HBV prevalence rate in Uganda should be interpreted with caution, since many of the Ugandan studies eligible for inclusion in our meta-analysis were conducted on HIV+ cohorts at the Infectious Diseases Institute (IDI) [13], at the Joint Clinical Research Council (JCRC) [71], under the Rakai Health Sciences project [5], or in the highly endemic northern region [6]. Furthermore, many of the Ugandan studies included were published between 2010 and 2015 – a period characterised by high prevalence of HBV in Uganda, with a national prevalence of 10% in the general population and a regional prevalence as high as 21.7% in the northeast and 19.4% in the north central region [72]. This explains the higher rate of HBsAg positivity in the general population. Most importantly, in Uganda, there have been no routine care programs for HBV-infected persons. Only recently were care programs initiated, such as routine testing, vaccination programs among high-risk groups such as health workers, viral load evaluation before treatment candidacy is established, and the Kabaka Foundation’s mass testing and vaccination program.

The pooled prevalence rate of HBV in Rwanda reported in our meta-analysis is analogous to the prevalence rate of 3.9% reported by the Rwanda national HBsAg prevalence survey [37]. Rwanda, unlike other EAC member countries, has published literature on the nationwide epidemiological survey of HBV infection [37] in addition to the sub-population studies, including studies of blood donors [28], persons living with HIV [43], health care workers [10], and pregnant women attending antenatal care [12, 26]. The low pooled prevalence of HBV in Rwanda compared to the other east African countries can be explained by the wide-scale vaccination campaign among people at high risk, on-going since 2015 [37].

Considering the year of publication, studies published between 2011 and 2015 reported a significantly higher HBV prevalence (8.759%) than those published from 2016 to 2020 (*P* < 0.0001). The dynamics underlying the periodic differences in the burden of the disease in our region can be explained by the variations in the prioritisation and target populations for HBV screening by the regional governments and funding organisations. Most of the studies conducted from 2010 to 2015 were targeting HBV-HIV co-infected persons. The apparently high burden of the disease in our region during this period can be explained by the shared route of transmission for both HIV and HBV [72]. The decline in the burden of the disease reported in the more recently published studies can be explained by the increased public health awareness and better vaccination coverage in our region [37]. Importantly, children born in health facilities after 2002 have been immunised against HBV as part of the expanded immunisation program contributing to herd immunity in our region [73]. Further still, publications from between 2016 and 2020 targeted participants from lower-risk groups, such as pregnant mothers attending antenatal care [39] and blood donors [28].

In terms of the screening techniques, the pooled prevalence rate obtained from published studies that had detected HBV using ELISA was significantly lower than that for studies that used RDT (*P* = 0.0126). This could be due to the higher specificity of ELISA compared to RDT [57]. Besides, RDTs have been found elsewhere to have sensitivity as low as 60%, despite being a diagnostic test of choice in community-based screening for infectious diseases (including HBV) due to their ease of use and simplicity [74].

In our meta-analysis of predictors of HBV infection, persons with a history of blood transfusion were 1.950 times more likely to be HBsAg-seropositive. This risk estimate was significantly higher than for persons without prior blood transfusion. This has been also reported in the meta-analysis of the HBV infection risk factors in Ghana [58]. Thus, despite the recommendation by the WHO to screen the donated blood for HBV prior to transfusion into the recipient [75], this is still not being done routinely in our region. Our meta-analysis indicates that blood should be screened rigorously before each blood transfusion to limit HBV infections.

Similarly, the pooled HBV prevalence in our meta-analysis was significantly higher in males. Men in east Africa are 1.312 times more likely than women to be HBsAg-positive, consistently with the results from primary studies conducted in west Africa [76]. This is probably because males are exposed to riskier sexual behaviours than females, like having multiple sexual partners [77]. Unfortunately, males have a low clearance rate of HBsAg, with eventual progression to chronic infection, increasing the risk of terminal liver diseases [78]. Our results therefore suggest that men should lead the fight against the spread of HBV, since they are at a much higher risk than women. Persons who have multiple sexual partners in our region also had significantly higher pooled HBV prevalence, and their chances of contracting HBV were 2.161 times higher than those of people with one sexual partner, consistently with findings from Nigeria [79] and India [80]. Furthermore, since HBV is a sexually transmitted disease, having multiple sexual partners increases the risk of infection. Our data suggest that sex education that promotes safe sex and good hygiene should be included in school programs and in public health awareness campaigns to reduce HBV infections. In addition, cultural and religious institutions should be brought on board to complement the school curricula and public health awareness programs in reducing promiscuity.

Furthermore, HBV prevalence was significantly higher among those who practice cultural scarification. This practice significantly increased the risk of HBV infection by 1.204, consistently with earlier reports from Sudan [81] and Ethiopia [82]. The increased risk can be explained by the use of poorly sterilised equipment during the practice. Consequently, mass sensitisation should be done to avoid cultural practices that pose a potential risk of HBV transmission. However, if the practices are deemed inevitable, as may be the case in some communities with strong ties to their cultures, a more professional approach should be adopted, such as use of sterilised or personalised equipment. Finally, HBV seropositivity was 2.227 times more likely to be linked with the risk of HIV co-infection. This is similar to findings reported from primary studies done is South Africa [83], Nigeria [84], Cameroon [85], Europe [86], and India [87]. The shared route of transmission of the two viruses is implicated in the increased risk of HBV infection among those infected with HIV [88]. Also, persons living with HIV (PLWHIV) have compromised immunity, and when they are exposed to HBV, the chances of HBV progressing to chronic infection of are higher [43]. Thus, rigorous screening of HIV-positive patients for the hepatitis B surface antigen should be introduced at lower health care facilities to assess the prevalence of co-infection in order to roll out customised treatment for co-infected persons. For the other predictors of HBV infection, including age, gravidity, education level, alcohol use, history of body piercing, and employment, findings from our meta-analysis show that host and viral factors appear to be more important in influencing the prevalence of HBV in the respective cohorts [89–91]. Worth mentioning is also that differences in vaccination coverage [64] or in health education regarding prevention of HBV transmission may account for the differences in the HBV prevalence between countries [65].

## Conclusion

In conclusion, this study has provided an update on the HBV status in our region. The studies included were robust; some had very large sample sizes, and they reflect the status quo of HBV in our region. Moreover, most of the studies were published recently (2015 to 2020) and offer up-to-date data on HBV prevalence in our region. Our meta-analysis has identified variations in HBV endemicity in east Africa, ranging from hyperendemicity to moderate endemicity. The overall pooled prevalence and the subgroup pooled prevalence are comparable to those observed in previous studies, with minor differences due to differences in endemicity and prevention strategies. Blood transfusion, surgery, body scarification, promiscuity, and gender were independent factors associated with the risk of HBV infection. Being male was singled out as the most highly significant factor associated with the HBV burden in our region. Consequently, persons in East Africa with a history of blood transfusion, surgery, and body scarification, persons with multiple sexual partners, and male persons had higher chances of hepatitis B surface antigen seropositivity. We recommend the implementation of universal and free vaccination for all adults in the region, as well as interventions by regional governments to control and prevent the transmission of HBV, targeting the higher-risk groups such as men.

## Supplementary Information

**Additional file 1: S1 Fig. A.** Forest plot of sub-group analysis of HBV prevalence for Uganda. **S2 Fig. B.** Forest plot of sub-group analysis of HBV prevalence for Kenya. **S3 Fig. C.** Forest plot of sub-group analysis of HBV prevalence for Rwanda. **S4 Fig. D.** Forest plot of sub-group analysis of HBV prevalence for Tanzania.

**Additional file 2: S5 Fig. E.** Forest plot of sub-group analysis of HBA prevalence in articles published from 2016 to 2020. **S6 Fig. F.** Forest plot of sub-group analysis of HBV prevalence in articles published from 2011 to 2015. **S7 Fig. G.** Forest plot of sub-group analysis of HBV prevalence in articles published from 2005 to 2010.

**Additional file 3: S8 Fig. H.** Forest plot of sub-group analysis of HBsAg detection by ELISA assay. **S9 Fig. I.** Forest plot of sub-group analysis of HBsAg detection by rapid diagnostic test (RDT) assay. **S10 Fig. J.** Forest plot of sub-group analysis of HBsAg detection by Enzyme Immune Assay (EIA). **S11 Fig. K.** Forest plot of sub-group analysis of HBsAg detection by other assays.

**Additional file 4: S 12 Table A.** Detailed Newcastle-Ottawa Scale for each included study in the meta-analysis.

## Data Availability

All data generated or analyzed during this study are included in this published article and as supplementary materials.

## Abbreviations

AJOL: African Journal Online
CLEIA: Chemiluminescent enzyme immunoassay
EAC: East African community
EIA: Enzyme Immuno Assay
ELISA: Enzyme Linked Immunosorbent Assay
FEM: Fixed effect model
HBcAb: Hepatitis B core antibody
HBeAb: Hepatitis B pre-core antibody
HBeAg: Hepatitis B pre-core antigen
HBsAb: Hepatitis B surface antibody
HBsAg: Hepatitis B surface antigen
HBV: Hepatitis B Virus
HIV: Human Immune Deficiency Virus
MEIA: Micro-particle Enzyme Immuno Assay
NOS: Newcastle-Ottawa Scale
QS: Quality assessment
RDT: Rapid diagnostic tests
REM: Random effect model
STIs: Sexually transmitted infections
WHO: World Health Organization
