# Supplementary material for: Prevalence and predictors of hepatitis B virus (HBV) infection in east Africa: evidence from a systematic review and meta-analysis of epidemiological studies published from 2005 to 2020
Source: Arch Public Health. 2021 Sep 18;79:167. doi: 10.1186/s13690-021-00686-1 (PMC8449462; doi:10.1186/s13690-021-00686-1)
Supplement: Supplementary file 1 — Additional file 1: S1 Fig. A. Forest plot of sub-group analysis of HBV prevalence for Uganda. S2 Fig. B. Forest plot of sub-group analysis of HBV prevalence for Kenya. S3 Fig. C. Forest plot of sub-group analysis of HBV prevalence for Rwanda. S4 Fig. D. Forest plot of sub-group analysis of HBV prevalence for Tanzania. [file 13690_2021_686_MOESM1_ESM.docx]

Supplementary materials S1-S4, Figures A-D


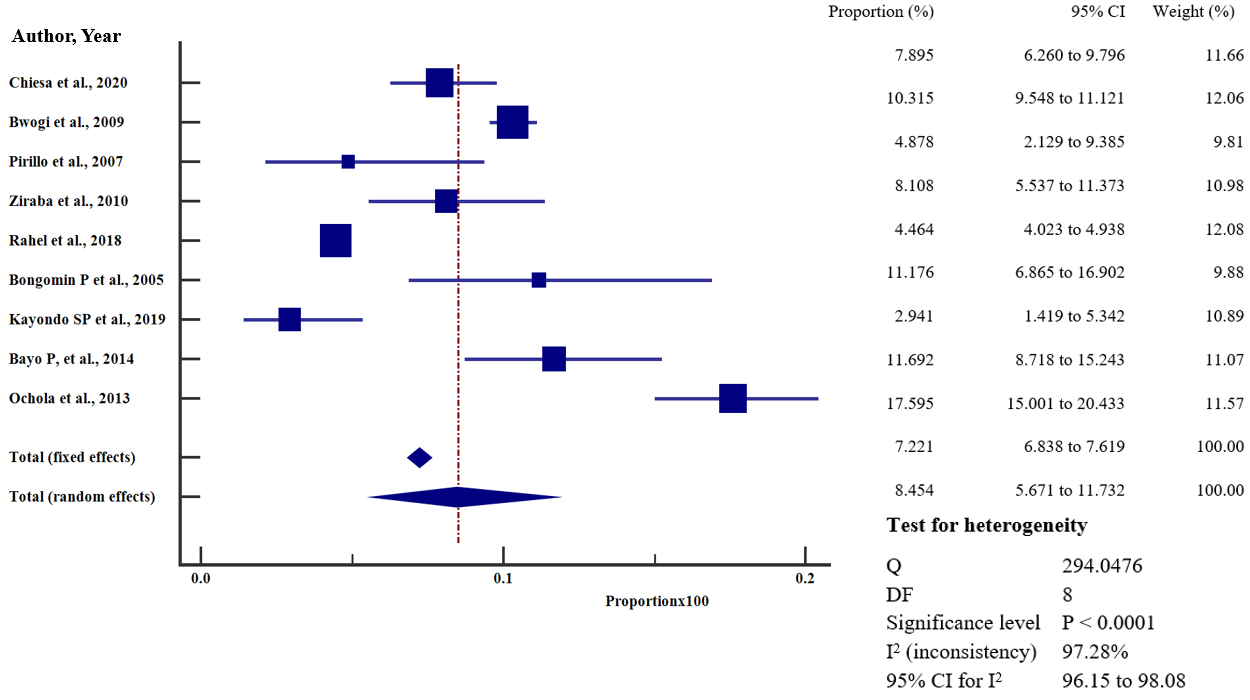


S1 Fig A. Forest plot of sub-group analysis of HBV prevalence for Uganda


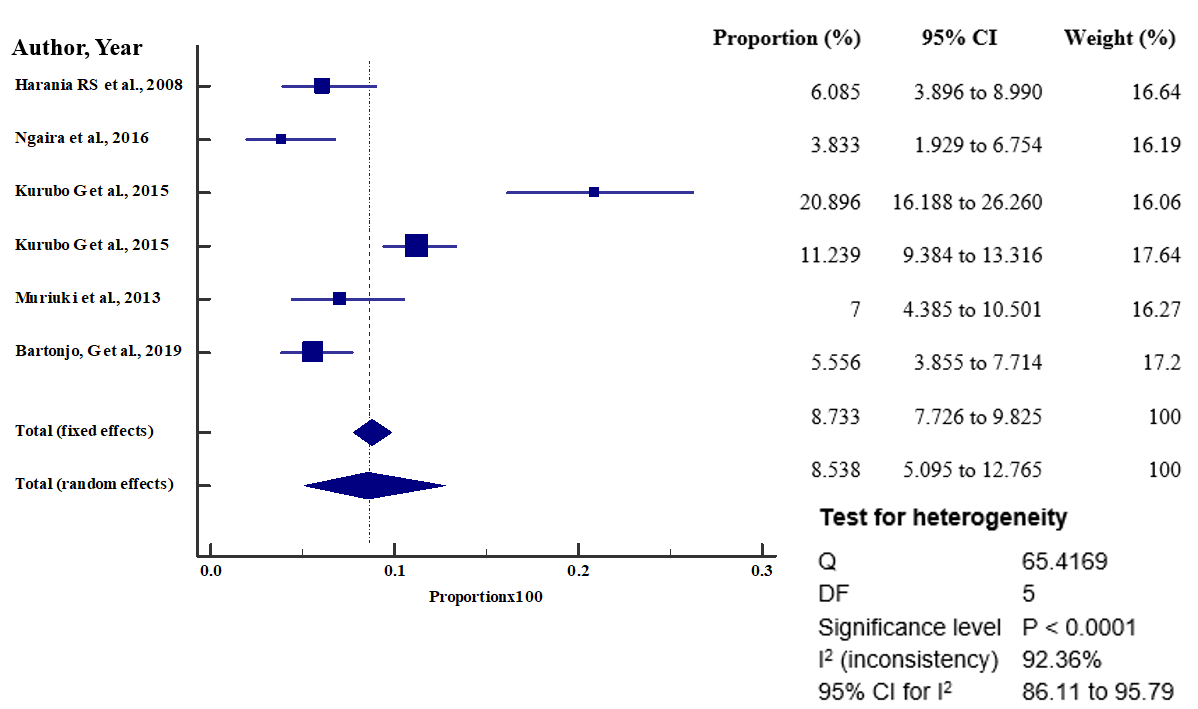


S2 Fig B. Forest plot of sub-group analysis of HBV prevalence for Kenya


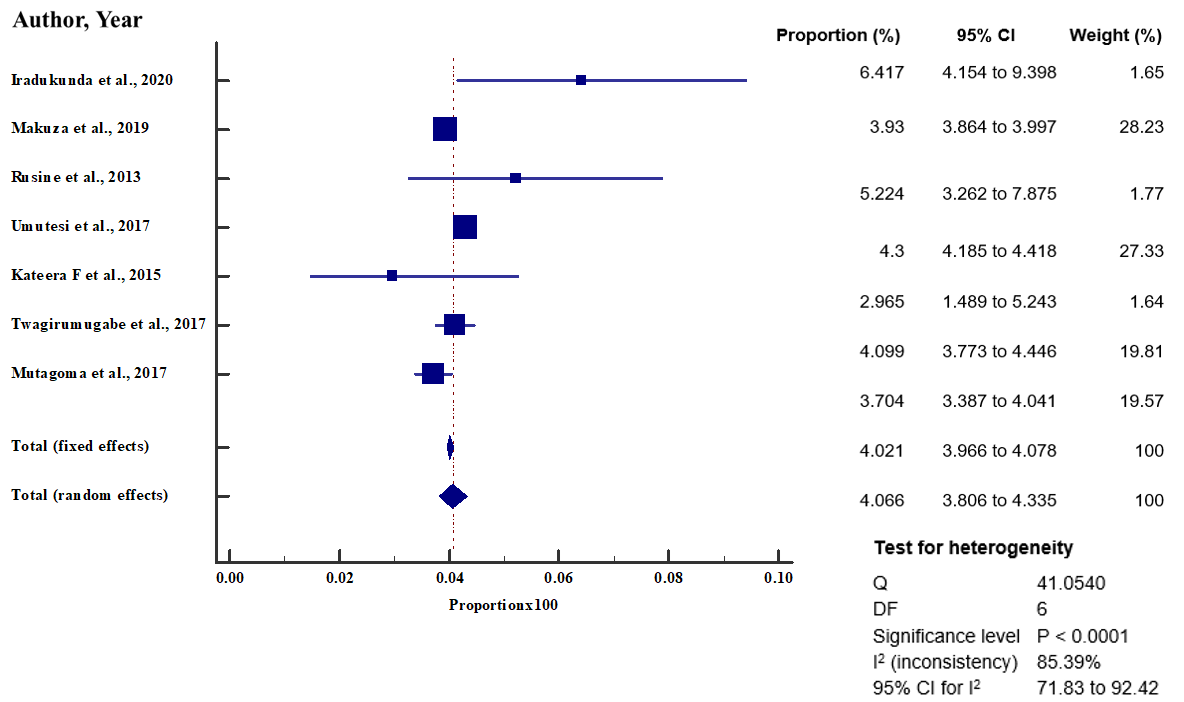


S3 Fig C: Forest plot of sub-group analysis of HBV prevalence for Rwanda


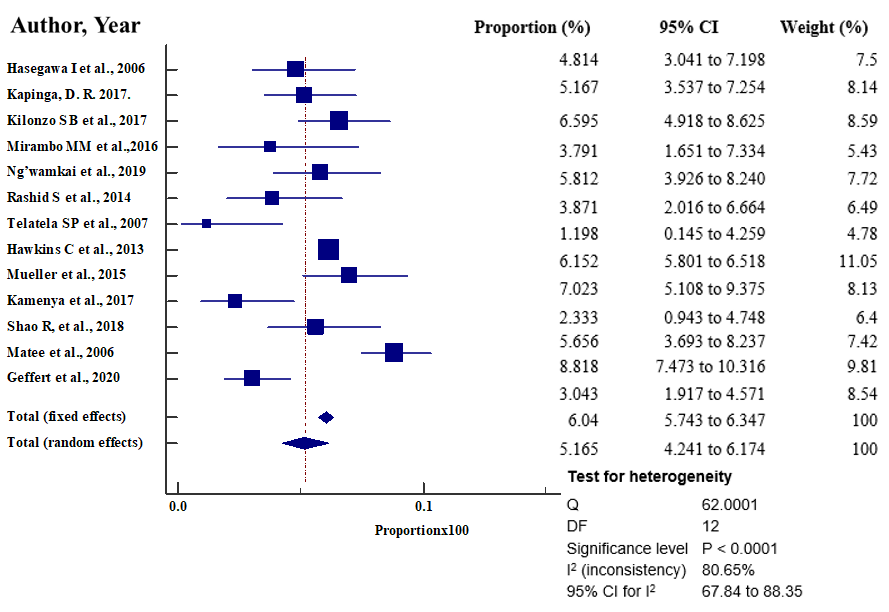


S4 Fig D. Forest plot of sub-group analysis of HBV prevalence for Tanzania
