# Supplementary material for: Prevalence and predictors of hepatitis B virus (HBV) infection in east Africa: evidence from a systematic review and meta-analysis of epidemiological studies published from 2005 to 2020
Source: Arch Public Health. 2021 Sep 18;79:167. doi: 10.1186/s13690-021-00686-1 (PMC8449462; doi:10.1186/s13690-021-00686-1)
Supplement: Supplementary file 2 — Additional file 2: S5 Fig. E. Forest plot of sub-group analysis of HBA prevalence in articles published from 2016 to 2020. S6 Fig. F. Forest plot of sub-group analysis of HBV prevalence in articles published from 2011 to 2015. S7 Fig. G. Forest plot of sub-group analysis of HBV prevalence in articles published from 2005 to 2010. [file 13690_2021_686_MOESM2_ESM.docx]

**Supplementary materials S5-S7, Figures E-G**


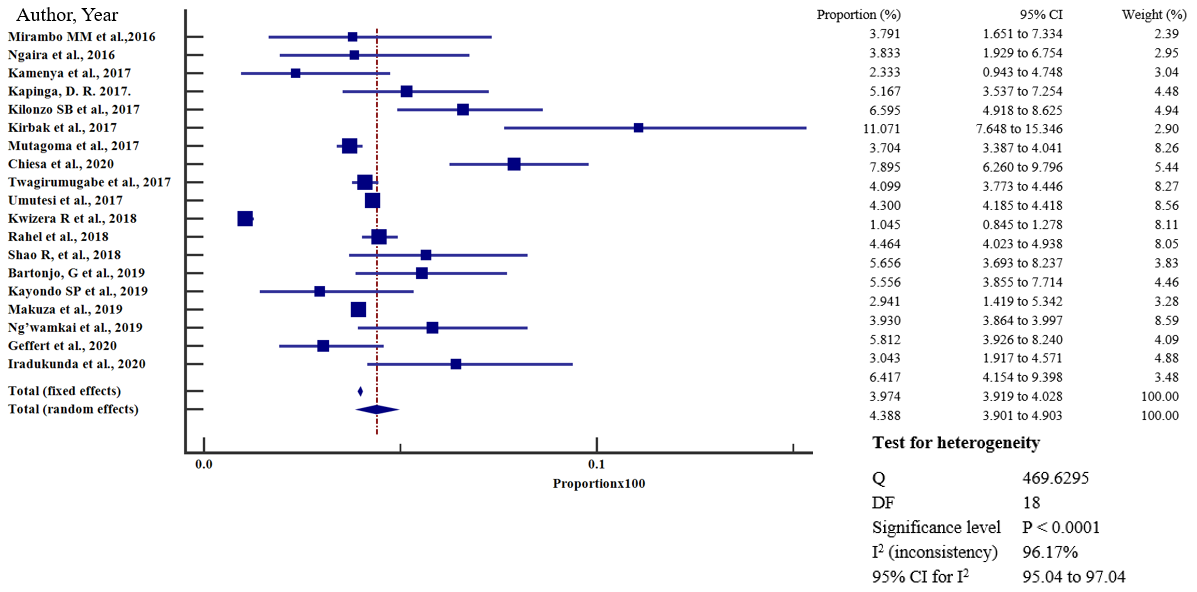


S5 Fig E. Forest plot of sub-group analysis of HBA prevalence in articles published from 2016-2020


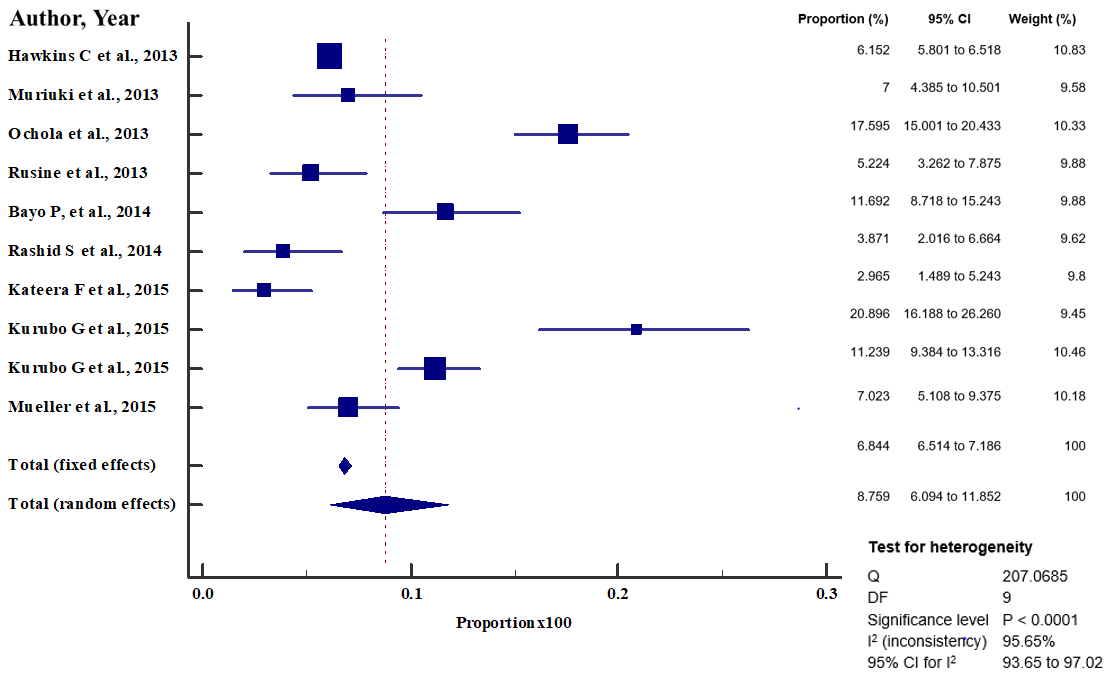


S6 Fig F. Forest plot of sub-group analysis of HBV prevalence in articles published from 2011-2015


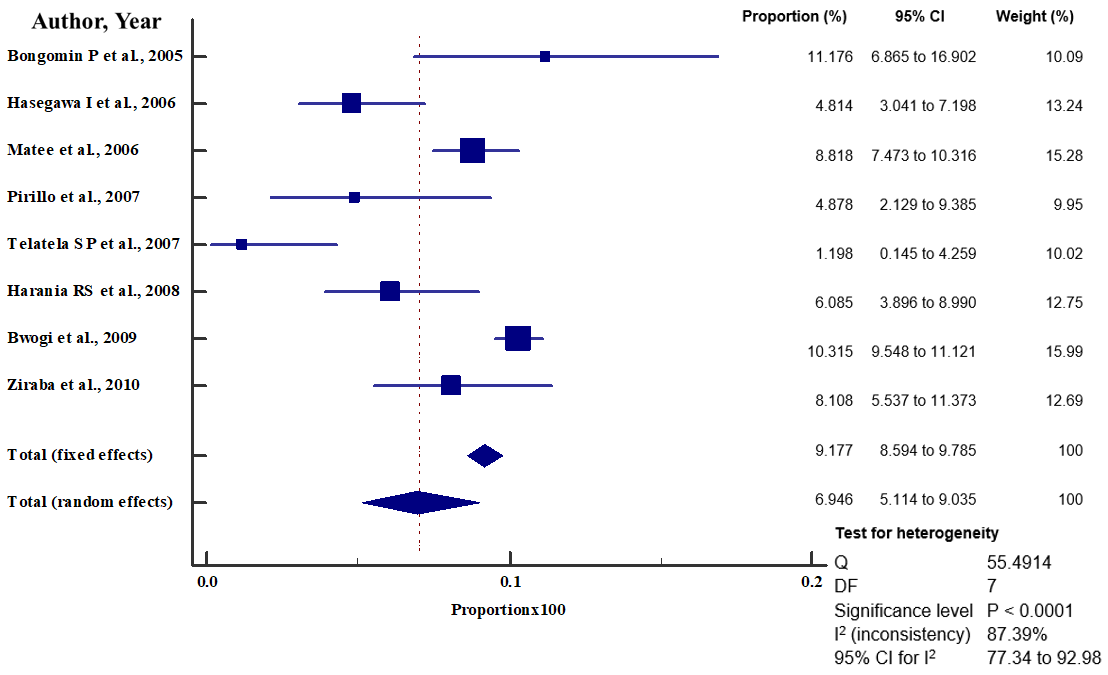


S7 Fig G. Forest plot of sub-group analysis of HBV prevalence in articles published from 2005-2010
