# Supplementary material for: Prevalence and predictors of hepatitis B virus (HBV) infection in east Africa: evidence from a systematic review and meta-analysis of epidemiological studies published from 2005 to 2020
Source: Arch Public Health. 2021 Sep 18;79:167. doi: 10.1186/s13690-021-00686-1 (PMC8449462; doi:10.1186/s13690-021-00686-1)
Supplement: Supplementary file 3 — Additional file 3: S8 Fig. H. Forest plot of sub-group analysis of HBsAg detection by ELISA assay. S9 Fig. I. Forest plot of sub-group analysis of HBsAg detection by rapid diagnostic test (RDT) assay. S10 Fig. J. Forest plot of sub-group analysis of HBsAg detection by Enzyme Immune Assay (EIA). S11 Fig. K. Forest plot of sub-group analysis of HBsAg detection by other assays. [file 13690_2021_686_MOESM3_ESM.docx]

**Supplementary materials S8-S11, Figures H-K**


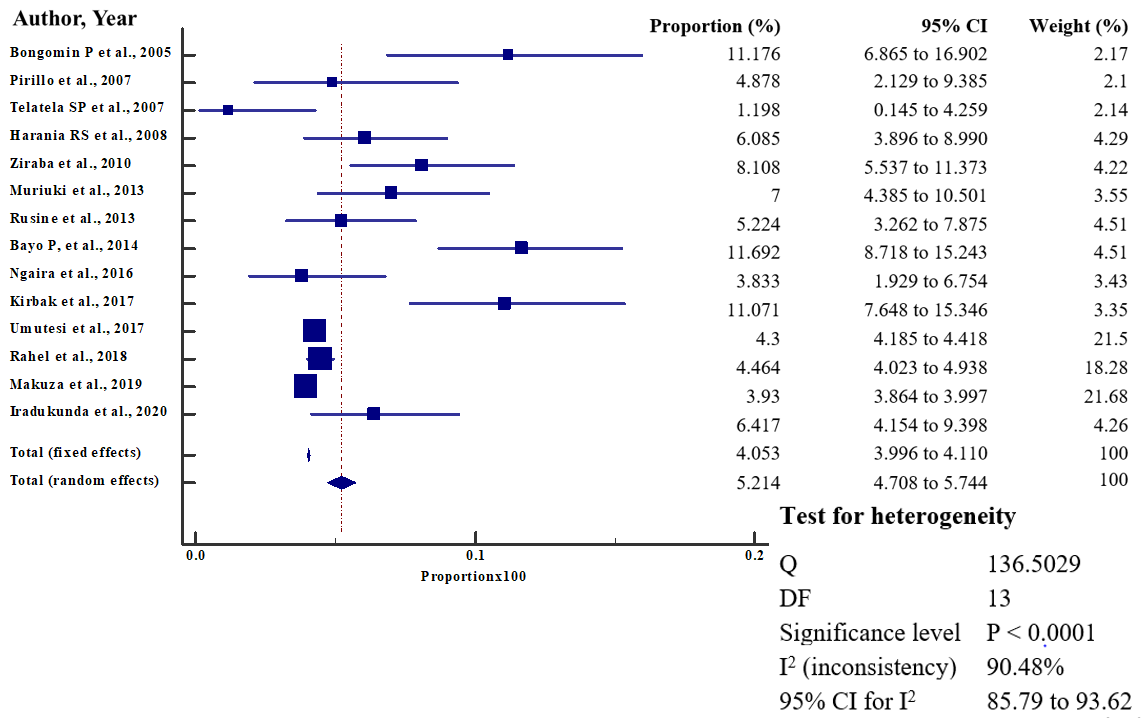


S8 Fig H. Forest plot of sub-group analysis of HBsAg detection by ELISA assay


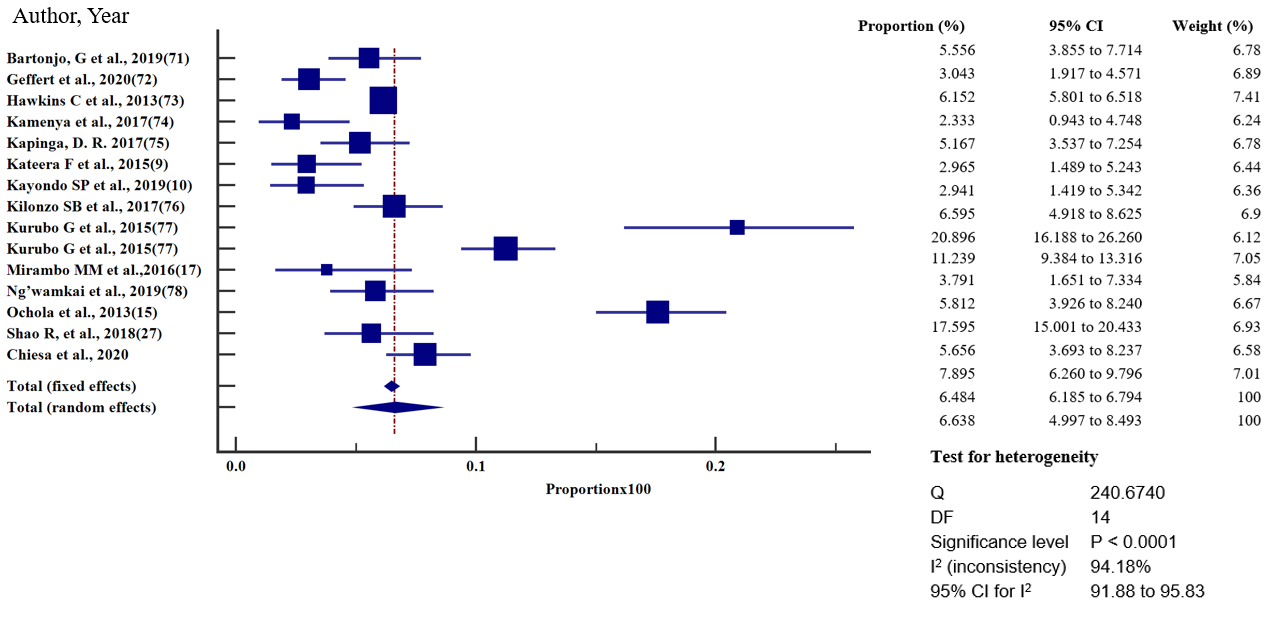


S9 Fig I. Forest plot of sub-group analysis of HBsAg detection by rapid diagnostic test (RDT) assay


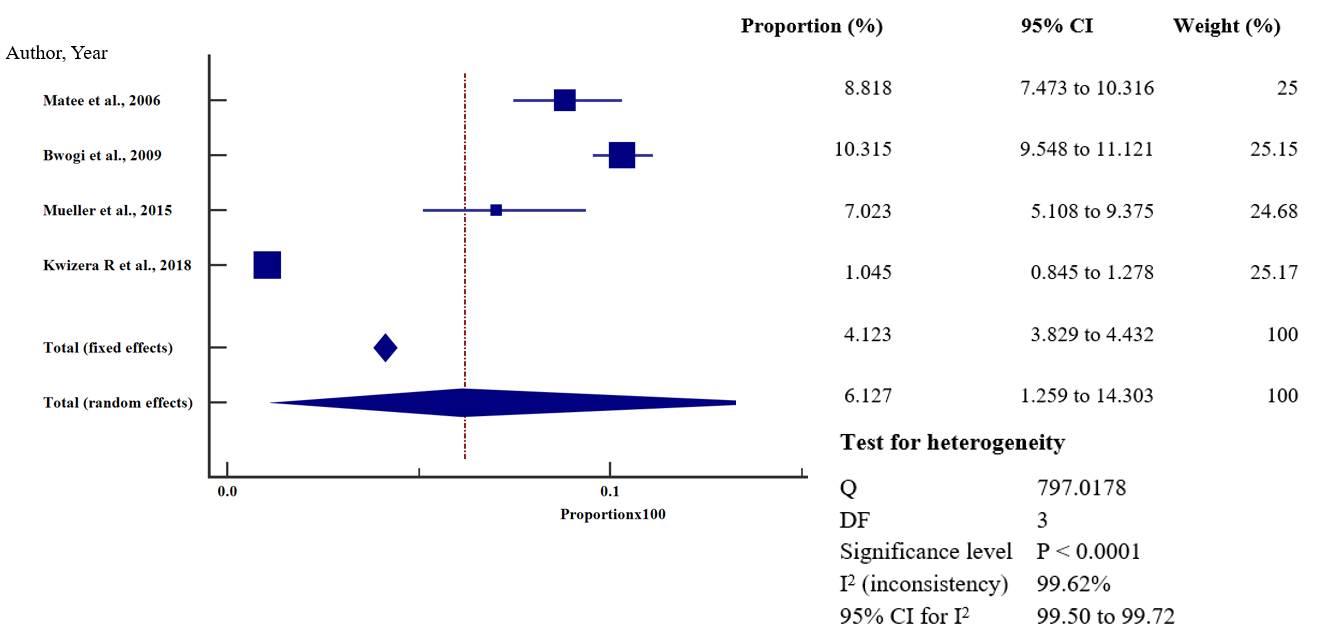


S10 Fig J. Forest plot of sub-group analysis of HBsAg detection by Enzyme Immune Assay (EIA)


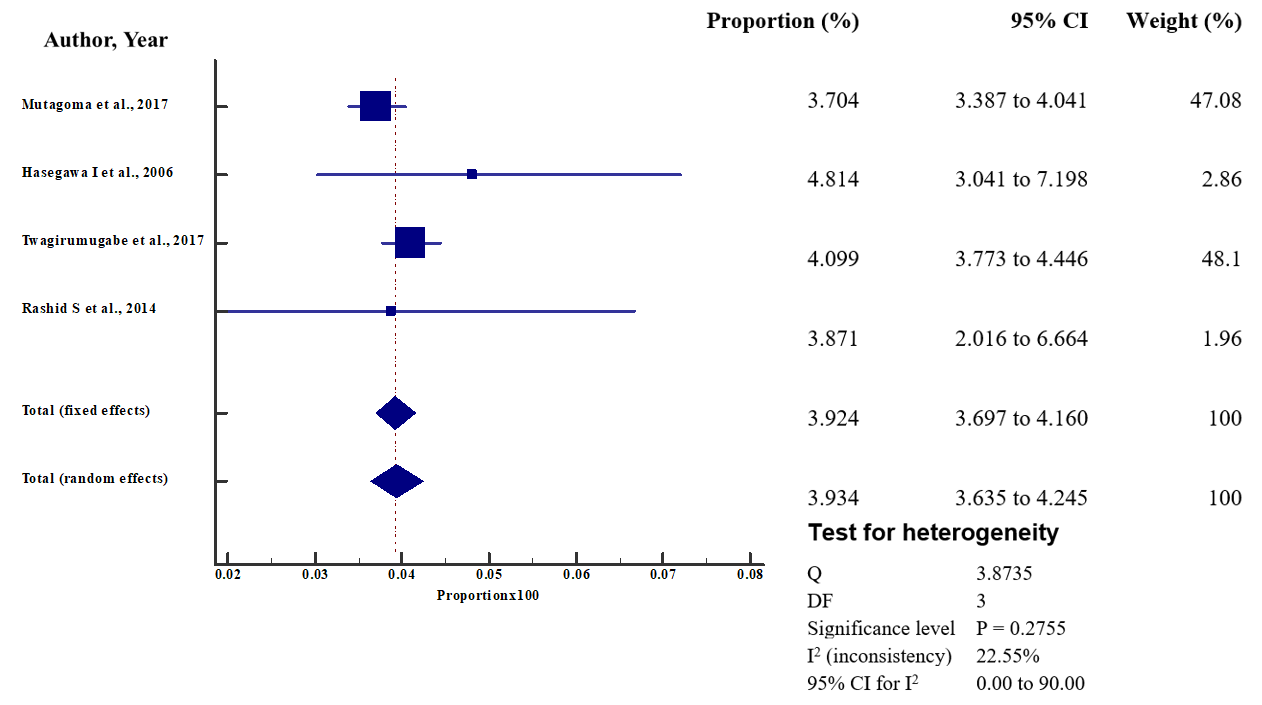


S11 Fig K. Forest plot of sub-group analysis of HBsAg detection by other assays
