# Supplementary material for: Prevalence and predictors of hepatitis B virus (HBV) infection in east Africa: evidence from a systematic review and meta-analysis of epidemiological studies published from 2005 to 2020
Source: Arch Public Health. 2021 Sep 18;79:167. doi: 10.1186/s13690-021-00686-1 (PMC8449462; doi:10.1186/s13690-021-00686-1)
Supplement: Supplementary file 4 — Additional file 4: S 12 Table A. Detailed Newcastle-Ottawa Scale for each included study in the meta-analysis. [file 13690_2021_686_MOESM4_ESM.docx]

S 12 Table A. Detailed Newcastle-Ottawa Scale for each included study in the meta-analysis.

| Study | Selection | | | | | Comparability | | Exposure | | Q S |
| --- | --- | --- | --- | --- | --- | --- | --- | --- | --- | --- |
|  | Representativeness of the sample | Sampling technique | Sample size | Inclusion criteria stated | Scope of the study defined | Assessed for risk  factors | Compared risk factors among study groups | Assessment of outcome | Statistical test |  |
| Bartonjo, G et al., 2019 | 0 | 1 | 1 | 1 | 1 | 1 | 1 | 1 | 1 | 8 |
| Bayo P, et al., 2014 | 1 | 1 | 1 | 1 | 1 | 1 | 1 | 1 | 1 | 9 |
| Bongomin P et al., 2005 | 0 | 0 | 1 | 1 | 1 | 0 | 0 | 1 | 1 | 5 |
| Bwogi et al., 2009 | 1 | 1 | 1 | 1 | 1 | 0 | 0 | 1 | 1 | 7 |
| Geffert et al., 2020 | 0 | 0 | 1 | 1 | 1 | 0 | 0 | 1 | 1 | 5 |
| Harania RS et al., 2008 | 0 | 0 | 1 | 1 | 1 | 0 | 0 | 1 | 1 | 5 |
| Hasegawa I et al., 2006 | 0 | 0 | 1 | 1 | 1 | 0 | 0 | 1 | 1 | 5 |
| Hawkins C et al., 2013 | 0 | 0 | 1 | 1 | 1 | 0 | 0 | 1 | 1 | 5 |
| Iradukunda et al., 2020 | 1 | 1 | 1 | 1 | 1 | 1 | 1 | 1 | 1 | 9 |
| Kamenya et al., 2017 | 0 | 0 | 1 | 1 | 1 | 0 | 0 | 1 | 1 | 5 |
| Kapinga, D. R. 2017. | 0 | 1 | 1 | 1 | 1 | 0 | 0 | 1 | 1 | 6 |
| Kateera F et al., 2015 | 0 | 0 | 1 | 1 | 1 | 1 | 1 | 1 | 1 | 7 |
| Kayondo SP et al., 2019 | 0 | 0 | 1 | 1 | 1 | 1 | 1 | 1 | 1 | 7 |
| Kilonzo SB et al., 2017 | 0 | 0 | 1 | 1 | 1 | 0 | 0 | 1 | 1 | 5 |
| Kirbak et al., 2017 | 0 | 1 | 0 | 1 | 1 | 0 | 0 | 1 | 1 | 5 |
| Kurubo G et al., 2015 | 0 | 0 | 0 | 1 | 1 | 1 | 1 | 1 | 1 | 6 |
| Kurubo G et al., 2015 | 1 | 0 | 1 | 1 | 1 | 1 | 1 | 1 | 1 | 8 |
| Kwizera R et al., 2018 | 1 | 0 | 1 | 1 | 1 | 1 | 1 | 1 | 1 | 8 |
| Makuza et al., 2019 | 0 | 1 | 1 | 1 | 1 | 1 | 1 | 1 | 1 | 8 |
| Matee et al., 2006 | 0 | 1 | 1 | 1 | 1 | 1 | 1 | 1 | 1 | 8 |
| Mirambo MM et al.,2016 | 0 | 0 | 0 | 1 | 1 | 1 | 1 | 1 | 1 | 6 |
| Mueller et al., 2015 | 0 | 0 | 1 | 1 | 1 | 0 | 0 | 1 | 1 | 5 |
| Muriuki et al., 2013 | 0 | 1 | 1 | 1 | 1 | 1 | 1 | 1 | 1 | 8 |
| Mutagoma et al., 2017 | 0 | 0 | 1 | 1 | 1 | 1 | 1 | 1 | 1 | 7 |
| Ng’wamkai et al., 2019 | 0 | 0 | 1 | 1 | 1 | 1 | 1 | 1 | 1 | 7 |
| Ngaira et al., 2016 | 0 | 0 | 1 | 1 | 1 | 1 | 1 | 1 | 1 | 7 |
| Ochola et al., 2013 | 1 | 1 | 1 | 1 | 1 | 0 | 0 | 1 | 1 | 7 |
| Pirillo et al., 2007 | 0 | 0 | 1 | 1 | 1 | 0 | 0 | 1 | 1 | 5 |
| Rachel et al., 2018 | 0 | 0 | 1 | 1 | 1 | 1 | 1 | 1 | 1 | 7 |
| Rashid S et al., 2014 | 0 | 0 | 1 | 1 | 1 | 0 | 0 | 1 | 1 | 5 |
| Rusine et al., 2013 | 0 | 0 | 1 | 1 | 1 | 1 | 1 | 1 | 1 | 7 |
| Seremba E, et al., 2017 | 0 | 0 | 1 | 1 | 1 | 1 | 1 | 1 | 1 | 7 |
| Shao R, et al., 2018 | 0 | 0 | 1 | 1 |  | 1 | 1 | 1 | 1 | 7 |
| Telatela SP et al., 2007 | 0 | 0 | 1 | 1 | 1 | 0 | 0 | 1 | 1 | 5 |
| Twagirumugabe et al., 2017 | 0 | 0 | 1 | 1 | 1 | 1 | 1 | 1 | 1 | 7 |
| Umutesi et al., 2017 | 0 | 0 | 1 | 1 | 1 | 0 | 0 | 1 | 1 | 5 |
| Ziraba et al., 2010 | 0 | 0 | 1 | 1 | 1 | 1 | 1 | 1 | 1 | 7 |
